# A Comprehensive Biophysical Model of Ion and Water Transport in Plant Roots. II. Clarifying the Roles of SOS1 in the Salt-Stress Response in *Arabidopsis*

**DOI:** 10.3389/fpls.2019.01121

**Published:** 2019-09-18

**Authors:** Kylie J. Foster, Stanley J. Miklavcic

**Affiliations:** Phenomics and Bioinformatics Research Centre, School of Information Technology and Mathematics Sciences, University of South Australia, Mawson Lakes, WA, Australia

**Keywords:** SOS1, Na^+^/H^+^ plasma membrane antiporters, Na^+^ transport, water transport, *Arabidopsis*, salt stress,osmotic stress, salt tolerance

## Abstract

SOS1 transporters play an essential role in plant salt tolerance. Although *SOS1* is known to encode a plasma membrane Na^+^/H^+^ antiporter, the transport mechanisms by which these transporters contribute to salt tolerance at the level of the whole root are unclear. Gene expression and flux measurements have provided conflicting evidence for the location of SOS1 transporter activity, making it difficult to determine their function. Whether SOS1 transporters load or unload Na^+^ from the root xylem transpiration stream is also disputed. To address these areas of contention, we applied a mathematical model to answer the question: what is the function of SOS1 transporters in salt-stressed *Arabidopsis* roots? We used our biophysical model of ion and water transport in a salt-stressed root to simulate a wide range of SOS1 transporter locations in a model *Arabidopsis* root, providing a level of detail that cannot currently be achieved by experimentation. We compared our simulations with available experimental data to find reasonable parameters for the model and to determine likely locations of SOS1 transporter activity. We found that SOS1 transporters are likely to be operating in at least one tissue of the outer mature root, in the mature stele, and in the epidermis of the root apex. SOS1 transporter activity in the mature outer root cells is essential to maintain low cytosolic Na^+^ levels in the root and also restricts the uptake of Na^+^ to the shoot. SOS1 transporters in the stele actively load Na^+^ into the xylem transpiration stream, enhancing the transport of Na^+^ and water to the shoot. SOS1 transporters acting in the apex restrict cytosolic Na^+^ concentrations in the apex but are unable to maintain low cytosolic Na^+^ levels in the mature root. Our findings suggest that targeted, tissue-specific overexpression or knockout of *SOS1* may lead to greater salt tolerance than has been achieved with constitutive gene changes. Tissue-specific changes to the expression of *SOS1* could be used to identify the appropriate balance between limiting Na^+^ uptake to the shoot while maintaining water uptake, potentially leading to enhancements in salt tolerance.

## Introduction

*SOS1* plays a critical, yet unclear, role in the salt tolerance of many plant species, ranging from glycophytes to halophytes. Reduction or knockout of *SOS1* expression resulted in salt sensitivity in tomato (Olías et al., 2009), *Arabidopsis thaliana* (Wu et al., 1996; Zhu et al., 1998), and the halophyte *Thellungiella salsuginea* (Oh et al., 2009), while overexpression of *SOS1* in transgenic *Arabidopsis* and *Chrysanthemum* led to enhanced salt tolerance (Shi et al., 2003; Yang et al., 2009; Gao et al., 2016). *SOS1* encodes a plasma membrane Na^+^/H^+^ antiporter, with transport through this antiporter driven by the pH gradient generated by plasma membrane H^+^-ATPases (Shi et al., 2000; Shi et al., 2002; Qiu et al., 2002). Despite the undeniable role of SOS1 in salt tolerance, how it actually contributes to this tolerance remains an open question (Munns and Tester, 2008; Britto and Kronzucker, 2015). A better understanding of the function of SOS1 could lead to the development of agriculturally important plant species with enhanced productivity under saline conditions.

Our understanding of the role of SOS1 in salt tolerance is hindered by uncertainty about its functional location in roots, especially in the mature root. *SOS1* expression analysis using promoter-GUS staining (Shi et al., 2002) combined with measurements of ^24^Na^+^ effluxes from the roots of *Arabidopsis* wild-type plants and *sos1* mutants (Hamam et al., 2016) suggests that SOS1 transporters do not contribute significantly to Na^+^ efflux out of the mature root; instead, they are responsible for Na^+^ exclusion from the apex. In contrast, more detailed spatial measurements of *SOS1* expression obtained using sectioning and microarray analysis (Brady et al., 2007; Winter et al., 2007)^1^, combined with vibrating microelectrode measurements of K^+^ and H^+^ fluxes in the mature root zone and apex of wild-type *Arabidopsis* plants and *sos1* mutants (Shabala et al., 2005), suggest that SOS1 contributes to Na^+^ efflux into the external medium along the whole root length, including the mature root. Therefore, while there is evidence that SOS1 is responsible for Na^+^ exclusion from the root apex (Shabala et al., 2005; Hamam et al., 2016), evidence for its role in the mature root is ambiguous and conflicting.

Na^+^ influx into root cells is passive, and SOS1 is the only known Na^+^ efflux protein acting on the plasma membrane of outer root cells (Plett and Møller, 2010; Shabala and Munns, 2017). If, as suggested by Shi et al. (2002) and Hamam et al. (2016), SOS1 is not acting in the mature outer root tissues, how is excessive accumulation of Na^+^ in the mature root cytosol prevented?

The role of SOS1 transporters in long distance Na^+^ transport is also controversial (Shabala and Munns, 2017). Based on the variable effects of *sos1* mutation on shoot Na^+^ content and the high level of expression of *SOS1* in the xylem parenchyma, Shi et al. (2002) proposed that SOS1 transporters remove Na^+^ from the xylem transpiration stream under severe salt stress but load Na^+^ under mild salt stress. However, as acknowledged by Shi et al. (2002), this proposal was speculative and direct experimental evidence was not available. In addition, this suggestion is countered by arguments based on the thermodynamics of Na^+^ transport *via* plasma membrane Na^+^/H^+^ antiporters, which instead point to these antiporters actively loading Na^+^ into the xylem transpiration stream (Munns and Tester, 2008; Shabala and Munns, 2017). However, the use of energy to increase Na^+^ transport toward the shoot is counterintuitive, and it is unclear how this process would contribute to salt tolerance.

Much of the uncertainty about SOS1 function stems from the need to infer physiological function from limited experimental data. To help shed light on this problem, we have taken a more direct approach and applied a biophysical model of ion and water transport in a salt-stressed plant root (Foster and Miklavcic, 2017) to answer the question: what functions do SOS1 transporters perform to enhance salt tolerance in plant roots? Our analysis proceeded in two stages. First, we optimized our model through quantitative comparisons between model predictions and existing experimental data on *Arabidopsis* roots. In the second phase, we refined our optimized model by simulating a range of specific locations of plasma membrane Na^+^/H^+^ antiporter activity. We analyzed how these different locations of SOS1 activity affected the transport of Na^+^ and water through the root—providing a wealth of physiological detail that is unattainable by experimental means. Since our model is based on *Arabidopsis*, our results are most relevant to the role of SOS1 in *Arabidopsis* roots.

We found that SOS1 transporter activity in the outer root tissues is essential to exclude Na^+^ from the root cytosol. In addition, we found that SOS1 transporters operating in the stele actively load Na^+^ into the xylem transpiration stream under all the external salt concentrations that we considered. Apart from increasing Na^+^ transport to the shoot, the loading of Na^+^ ensures increased water transport to the shoot to counteract the osmotic stress associated with highly saline soil conditions.

## Method

### Model Description

**Figure 1:**
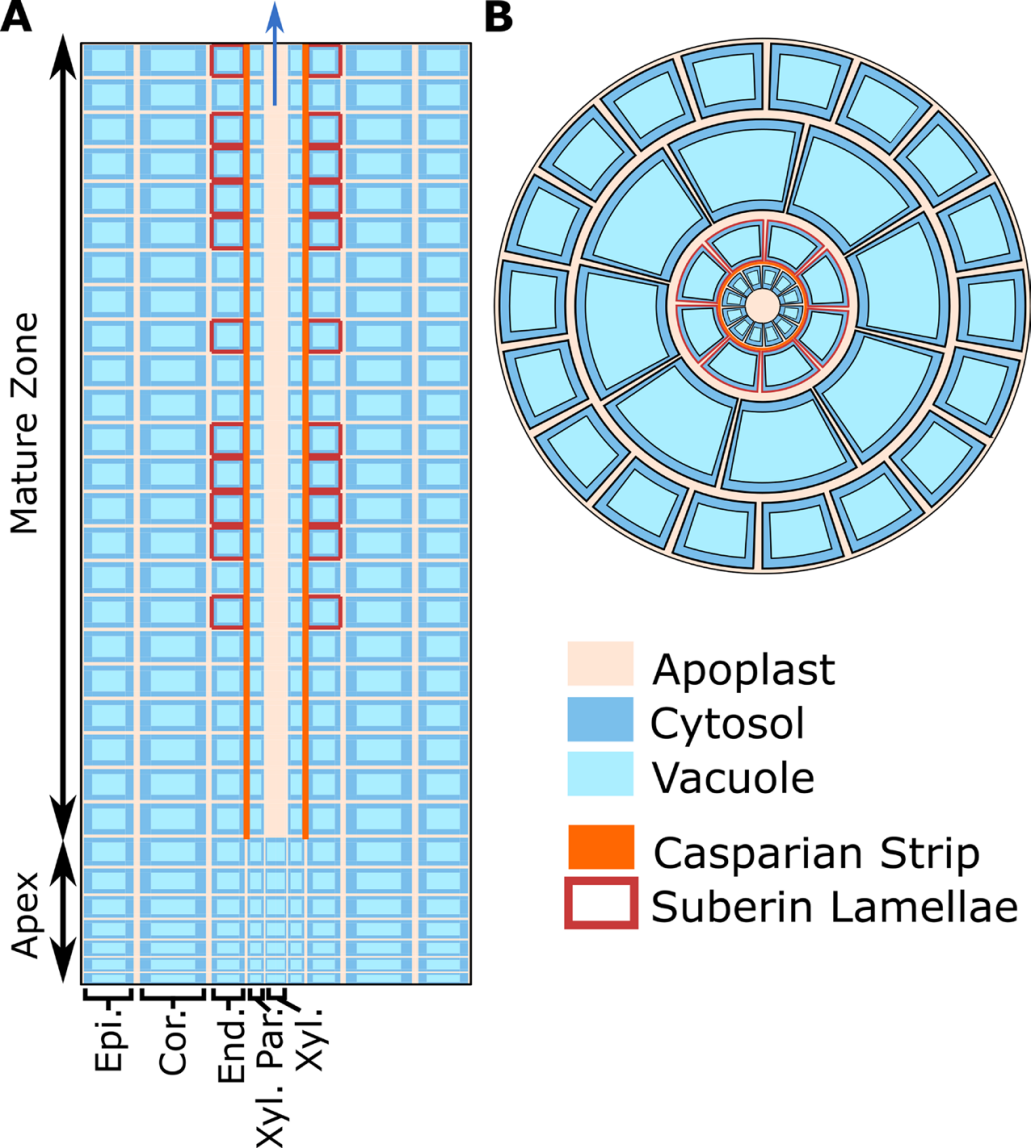
Cross-sections of the model root cylinder. **(A)** Longitudinal cross-section of the root showing the two developmental zones (apex and mature root) and the endodermal barriers (Casparian strip and suberin lamellae). **(B)** Top view of the root showing that each tissue type contains multiple cells and is divided into apoplastic, cytosolic, and vacuolar compartments. The model root is divided in the axial and radial directions to create annular cylinders that are the height and width of a single cell. Each annular cylinder represents a single cell high ring of cells of a specific type: epidermis (Epi.), cortex (Cor.), endodermis (End.), xylem parenchyma (Xyl. Par.), and xylem (Xyl.). Although not shown here, plasmodesmata connect all root cytosols in the model. Adapted from Foster and Miklavcic (2017).

**Figure 2:**
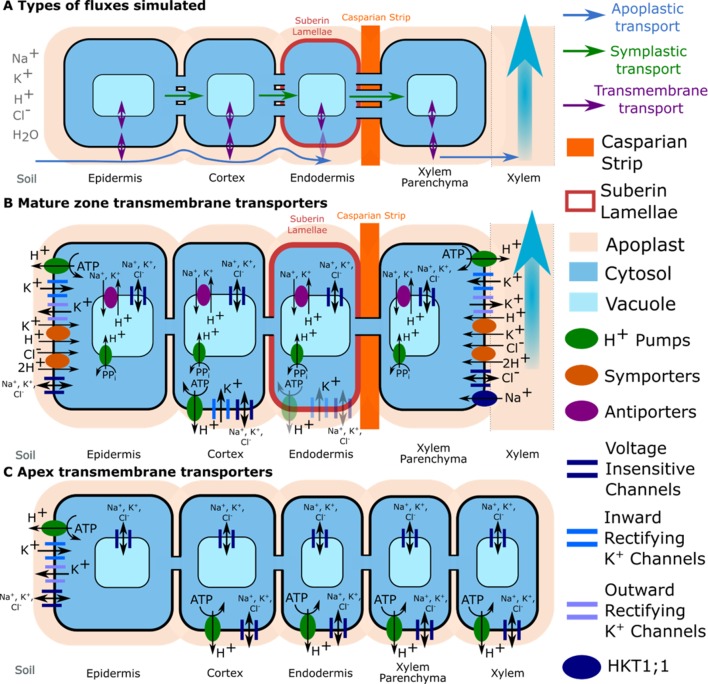
Transport pathways and membrane transport proteins included in the model. **(A)** Close-up of a single layer of cells in the mature zone highlighting the different transport pathways simulated for both ions and water (apoplastic, symplastic, and transmembrane). Axial symplastic and apoplastic fluxes are also simulated; although for visual clarity, they are not included in the figure. **(B)** Location of transporter and channel activity in the mature root. **(C)** Location of transporter and channel activity in the apex. The distribution of K^+^ voltage–dependent channels and K^+^/H^+^ symporters were obtained from the literature: *Arabidopsis* eFP Browser, Lagarde et al. (1996), Gaymard et al. (1998), Ivashikina et al. (2001), Desbrosses et al. (2003), and Gierth et al. (2005). Tonoplast Na^+^/H^+^ antiporters were assumed to be present in all cells in the mature zone, but not the apex (Shi et al., 2002). Trans-plasma membrane transport in the mature endodermis was assumed to occur only in cells lacking suberin lamellae (e.g., passage cells). Plasma membrane Na^+^/H^+^ antiporters are not shown because a wide range of spatial distributions were considered (see the section *Investigating Possible SOS1 Location and Resulting Function*). Arrows indicate the typical direction of transport identified during the model simulation.

As a basis for this study, we used our previous model of ion and water transport in a salt-stressed root (Foster and Miklavcic, 2017)—with some important modifications—to investigate the location and function of SOS1. The model combines the features of our single cell, active and passive, membrane transport models (Foster and Miklavcic, 2015) with the structure of our earlier models of passive transport in a plant root (Foster and Miklavcic, 2013; Foster and Miklavcic, 2014; Foster and Miklavcic, 2016). A schematic of the model root is shown in **Figure 1**, while the transport proteins included in the model and their assumed spatial distributions are summarized in **Figure 2**. The root is divided into two developmental zones: a mature zone, which includes functional xylem; the Casparian strip and suberin lamellae; and an apex, which does not contain these features (see **Figure 1A**). The structure of the root and the transport parameters were chosen to represent *Arabidopsis* roots due to their relatively simple geometry and the availability of suitable experimental data.

The model simulates the radial and axial transport of Na^+^, K^+^, Cl^–^, H^+^, and water through the root apoplast and symplast (*via* plasmodesmata), as well as across plasma membranes and tonoplasts *via* passive channels and active transporters. It includes trans-plasma membrane transport *via* H^+^ pumps, Na^+^/H^+^ antiporters, K^+^/H^+^ and Cl^–^/2H^+^ symporters, voltage-insensitive channels, inward rectifying K^+^ channels, outward rectifying K^+^ channels and HKT1;1, as well as trans-tonoplast transport *via* H^+^ pump, Na^+^/H^+^, and K^+^/H^+^ antiporters and channels. The model includes fixed anionic charges in the apoplast and H^+^ buffering in cytosols and vacuoles. The model equations are summarized in the **Supplementary Material**. Details about the underlying model assumptions are provided in Foster and Miklavcic (2015, 2017).

Ion transport through the apoplast and the symplast is governed by electrochemical diffusion and convection and is simulated using an extended Nernst–Planck equation. Electric potentials are simulated by assuming local electroneutrality. Water flow, which is modeled using nonequilibrium thermodynamics, is driven by: both hydraulic and osmotic pressure gradients for transport across plasma membranes and through the symplast, osmotic pressure gradients only for transport across tonoplast membranes, and hydraulic pressure gradients only for transport *via* the apoplast. Ion transport through pumps and symporters is modeled using four state carrier cycles (see **Figure S1**), while transport through antiporters is modeled using the law of mass action (Foster and Miklavcic, 2015). Transport through channels is modeled using the Goldman–Hodgkin–Katz current equation, with voltage gating represented by Boltzmann distributions assuming two state channels—open/closed (Foster and Miklavcic, 2015).

In this study, the following extensions have been made to the basic Foster and Miklavcic (2017) model: the inclusion of Na^+^ transport *via* HKT1;1 in the stele (Xue et al., 2011), the addition of apoplastic K^+^ concentration dependence for the voltage gating of outward rectifying K^+^ channels (Ivashikina et al., 2001; Johansson et al., 2006), and the inclusion of external Ca^+2^ concentration dependence for the plasma membrane nonselective cation channel permeabilities (Demidchik and Tester, 2002). Details are provided in the **Supplementary Material**. To compensate for the added complexity, while still allowing reasonable simulation time, the length of the root was reduced from 50 to 30 cells to allow for many scenarios to be simulated in a timely manner.

The resulting non-linear system of coupled differential algebraic equations was solved numerically in MATLAB^®^ using the ode15s package (Foster and Miklavcic, 2017). All simulations assumed a hydraulic pressure boundary condition of −0.3 MPa at the top of the root.

### Biophysical Model Optimization

The model system was optimized on the basis of existing experimental data for *Arabidopsis* wild-type plants and *sos1* mutants (where possible) including: root Na^+^ and K^+^ content (Davenport et al., 2007; Mason et al., 2010); ^22^Na^+^ tracer fluxes from the root to the shoot (Davenport et al., 2007); xylem Na^+^, K^+^, and anion concentrations in intact plants (Hall et al., 2006); and epidermal transmembrane potentials (Shabala et al., 2005). As Cl^–^ is the only mobile anion assumed in our model, the predicted Cl^–^ concentrations were compared with the *total* measured concentrations of the most significant monovalent anions, Cl^–^ plus $NO_{3}^{-}$. We did not use radioactive tracer fluxes across the root surface because of the uncertainty about their accuracy (Malagoli et al., 2008; Britto and Kronzucker, 2015; Flam-Shepherd et al., 2018; Munns et al., 2019b). We used root ion contents measured on a fresh weight basis since comparisons with dry weight root contents are significantly affected by the assumed moisture content.

Since the model provides a working approximation to the predominant transport processes known to occur in real roots, we did not expect to achieve complete agreement between model outcomes and experimental data through this optimization process. Instead, the optimization determines the most reasonable model parameter estimates that are consistent with experiment. These values are indicative of the location and function of SOS1 transporters; they are also used to explore physiological behavior in more detail than is possible to achieve experimentally.

**Figure 3:**
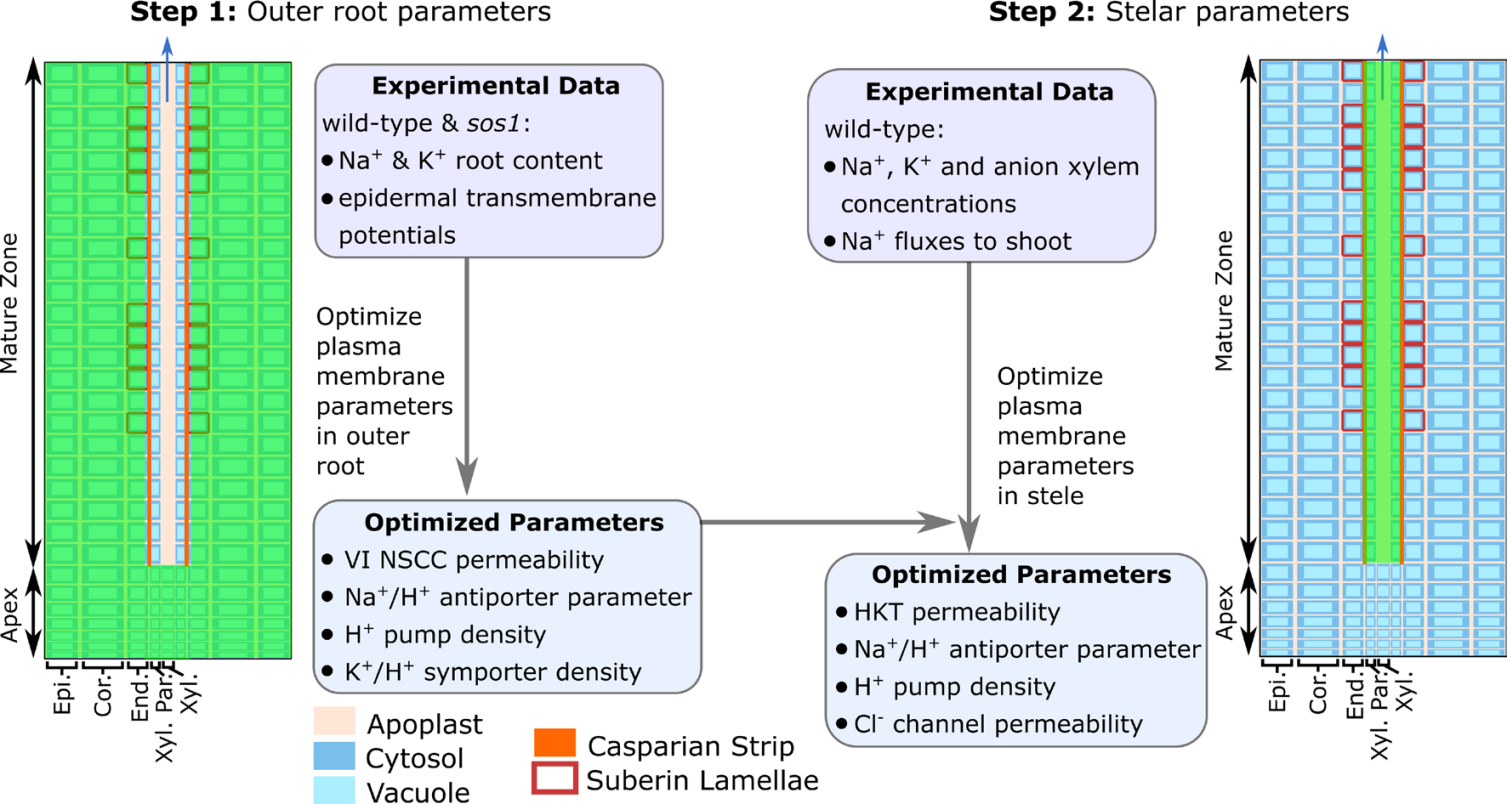
The two-stage procedure used to optimize the model parameters. The green shading indicates the root region in which the parameters were optimized in each stage. VI NSCC refers to voltage-insensitive nonselective cation channels.

We found that transport processes in the outer root tissues influenced *all* of the quantities that were utilized in the model optimization, while the transport processes in the stele influenced only the predicted xylem concentrations and fluxes. Taking advantage of this, we optimized the model in two stages (see **Figure 3**). First, we used the experimentally measured root ion contents and epidermal membrane potentials to optimize the system parameters controlling the transport processes in the outer root tissues. Second, we used the resulting optimized outer root parameters, combined with experimentally measured xylem ion concentrations and ^22^Na^+^ tracer fluxes from the root to the shoot, to optimize the system parameters controlling the stellar processes.

Due to the complexity of the model and the limited experimental data, it was not feasible to optimize all of the model parameters. Instead, we used a parameter sensitivity analysis to determine those parameters that had the greatest influence on the experimental variables and employed only these in the optimization procedure (see **Figure 3** and **Table S1**). These parameters were optimized in MATLAB^®^ using fmincon. The remaining parameters and their sources are summarized in **Table S2**.

### Investigating Possible SOS1 Location and Resulting Function

For the model optimization described above, we assumed SOS1 transporters were operating in all tissues, and we allowed the governing transport parameters to vary between the outer root tissues and the stele. To address in more detail the questions of where SOS1 transporters are operating and their subsequent functions, in the second stage of our study, we manually explored a wider range of spatial distributions and transport strengths for the plasma membrane Na^+^/H^+^ antiporters. We examined the effects of these antiporters operating according to the following scenarios:

- in individual tissue regions in the mature zone: the epidermis, cortex, endodermis, and xylem parenchyma;
- in individual tissue regions in the apex: the epidermis, cortex, endodermis, xylem parenchyma, and non-functional xylem;
- in combinations of tissues in the mature zone: the epidermis and the xylem parenchyma, the cortex and the xylem parenchyma, all outer tissues, and all tissues in the mature zone;
- in combinations of tissues in the mature zone and apex: the apical epidermis and the mature xylem parenchyma, and all apex tissues and the mature xylem parenchyma;
- in all tissues in the apex; and, finally,
- in all root tissues.

For the mature zone scenarios, the plasma membrane antiporters were assumed to be absent from the apex, and conversely, for the apex scenarios, the plasma membrane antiporters were assumed to be absent from the mature zone. The following wide range of antiporter parameter values was considered for each spatial distribution scenario: 1, 10, 100, and 1,000×10^–8^ m^4^ mol^–1^ s^–1^. This analysis provided a finer level of detail than can be achieved experimentally.

## Results

### Biophysical Model Optimization

**Figure 4:**
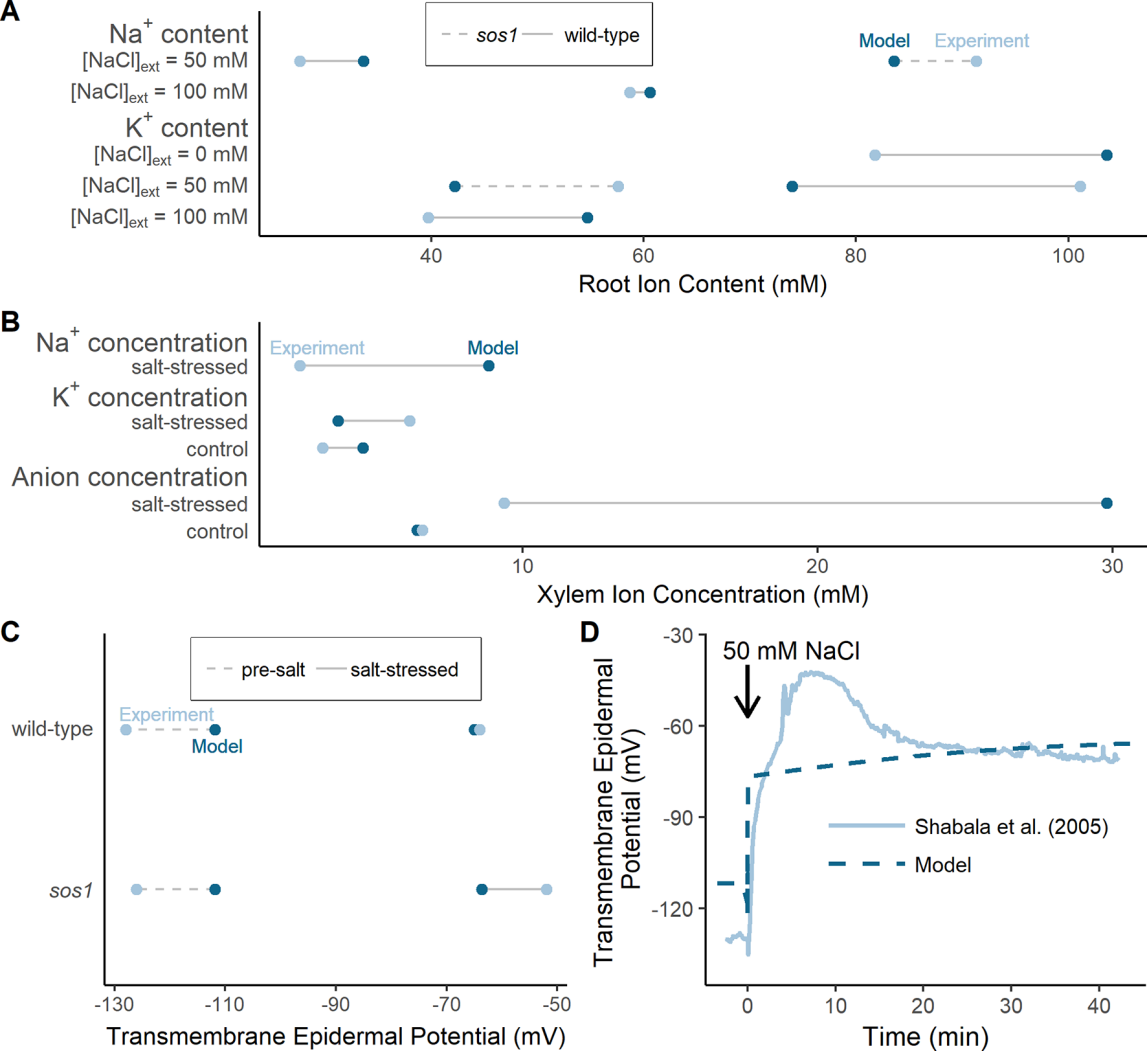
The optimized model simulations are comparable to a wide range of experimental measurements. **(A)** Comparison of optimized model (dark blue circles) and experimental (light blue circles) root Na^+^ and K^+^ content measurements, for external NaCl concentrations ranging from 0 to 100 mM, for wild-type plants (solid lines) and *sos1* mutants (dashed lines). **(B)** Comparison of optimized model (dark blue circles) and experimental (light blue circles) xylem concentrations of Na^+^, K^+^, and anions, in the presence and absence of 50 mM NaCl. **(C)** Comparison of optimized model (dark blue circles) and experimental (light blue circles) pre-salt (dashed lines) and salt-stressed (solid lines) transmembrane epidermal potentials. **(D)** Comparison of optimized model (dashed dark blue line) and experimental (solid light blue line) changes in transmembrane epidermal potential over time. All simulations were conducted using the parameters in **Tables S1** and **S2**. Experimental data was obtained from Shabala et al. (2005), Hall et al. (2006), Davenport et al. (2007) and Mason et al. (2010). Comparisons with: Davenport et al. (2007) were conducted with 50 mM NaCl, 11.9 mM KCl, 0.5 mM Ca^2+^, and a pH of 5.5 in the external medium; Hall et al. (2006) were conducted with 50 mM NaCl, 2 mM KCl, 2 mM Ca^2+^, and a pH of 4.4 in the external medium; Mason et al. (2010) were conducted with 100 mM NaCl, 7.5 mM KCl, 0.8 mM Ca^2+^, and a pH of 5.7 in the external medium; Shabala et al. (2005) were conducted with 50 mM NaCl, 0.5 mM KCl, 0.1 mM Ca^2+^, and a pH of 5.5 in the external medium.

The results of the model optimization are summarized in **Figure 4** and **Table 1** (see **Table S1** in the **Supplementary Material** for associated optimized parameter values). While perfect agreement with experiment cannot be expected, it is encouraging to see that the optimized model results are all of the same order of magnitude as the experimental measurements. This indicates that the model has captured the general function of the dominant processes. The Na^+^ flux to the shoot (**Table 1**), the salt-stressed epidermal transmembrane potentials (**Figures 4C, D**), the control anion concentrations in the xylem (**Figure 4B**), and the Na^+^ root contents (**Figure 4A**) match the experimental data particularly well. The comparison also points to possible mechanisms that are not represented. For example, one of the largest differences between the optimized model predictions and the experimental observations occurred with the concentration of anions in the xylem under salt-stressed conditions; the model anion concentration is approximately three times larger than the experimentally determined concentration (**Figure 4B**). This difference is discussed in the section *Discussion*.

**Table 1:** Experimentally measured and optimized model Na^+^ fluxes to shoot.

Source	Na^+^ flux to shoot (nmol min g root FW)
Davenport et al. (2007)	58
Optimized model	50

Simulations were conducted with 50 mM NaCl, 1.9 mM KCl, 0.5 mM Ca^2+^, and a pH of 5.5 in the external medium, using the parameters in **Tables S1** and **S2**.

The optimized plasma membrane Na^+^/H^+^ antiporter strengths in the outer root tissues and in the stele were found to be similar (2 × 10^–7^ and 3 × 10^–7^ m^4^ mol^–1^ s^–1^, respectively; see **Table S1**), which suggests that these antiporters operate at a similar rate in the inner and outer root tissues. We examine the possible spatial distributions of SOS1 transporters in more detail in the following sections.

What is also of possible interest is the finding (**Table S1**) that a *low*, as opposed to *no*, level of plasma membrane Na^+^/H^+^ antiporter activity was required for the *sos1* scenarios shown in **Figure 4**. Assuming *zero* antiporter activity for the *sos1* mutant scenario was insufficient to achieve reasonable agreement between the model predictions and the experimental *sos1* measurements, even allowing for a wide range of passive Na^+^ permeabilities to compensate (see **Figure S2** in the **Supplementary Material**).

### Where are SOS1 Transporters Operating in roots?

**Figure 5:**
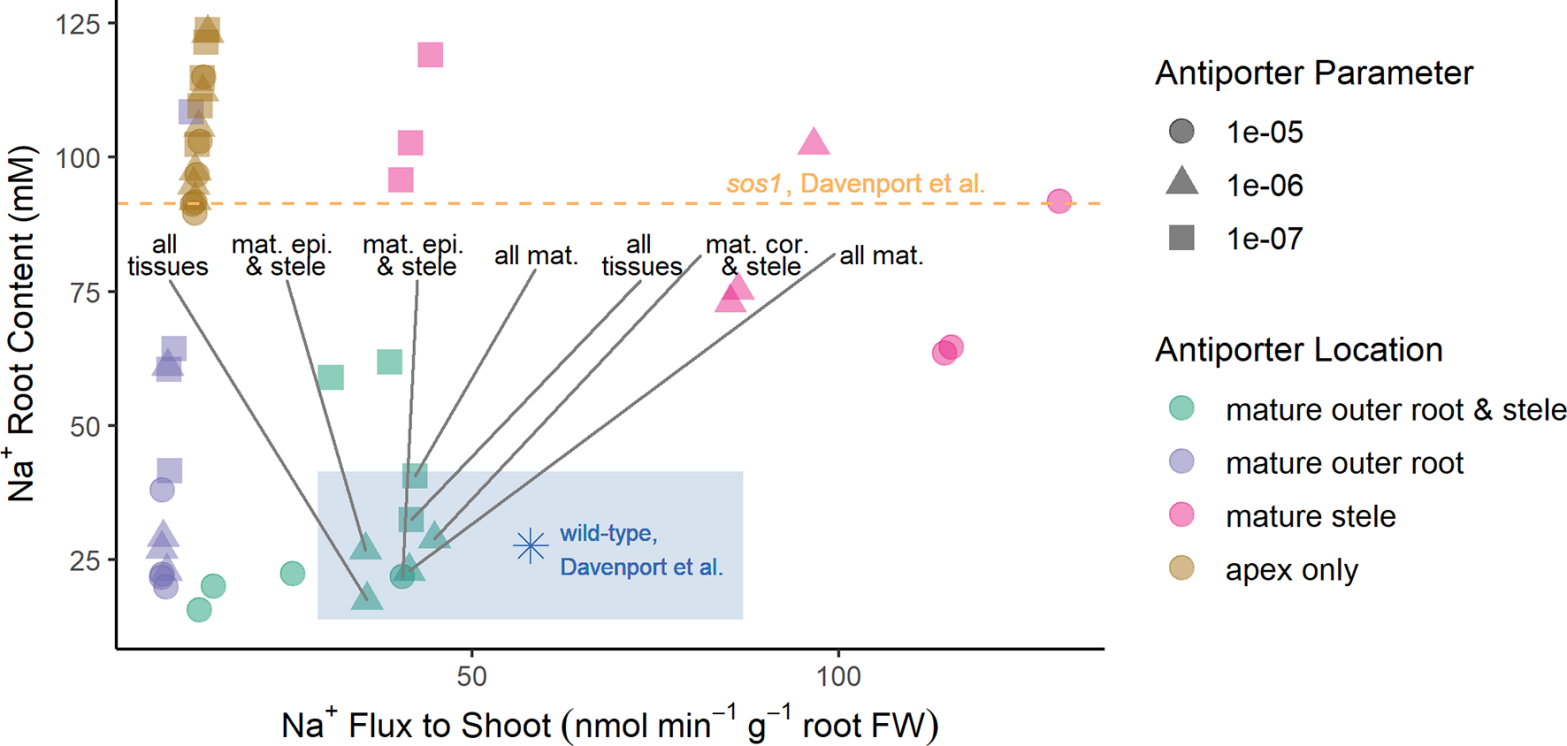
Reasonable Na^+^ root contents and Na^+^ fluxes to the shoot require plasma membrane Na^+^/H^+^ antiporters to be operating in both the mature outer root tissues and the stele. A wide range of spatial distributions were considered for the plasma membrane Na^+^/H^+^ antiporters. These are displayed in four categories: antiporters operating in at least the mature outer root tissues and stele (green); antiporters operating in at least the mature outer root, but not the stele (purple); antiporters operating in at least the mature stele, but not the mature outer root tissues (pink); and antiporters operating in only the apex (brown). The blue asterisk and orange dashed line indicate experimentally measured values (Davenport et al., 2007) for wild-type *Arabidopsis* and *sos1* roots, respectively. The blue-shaded region indicates reasonable values of Na^+^ root content and Na^+^ fluxes to the shoot based on the measurements of Davenport et al. (2007). Scenarios identified as reasonable are labeled; they include antiporters operating in: all tissues, all mature tissues, the mature epidermis and stele, and the mature cortex and stele. A wide range of plasma membrane Na^+^/H^+^ antiporter parameter values were considered: 10^–5^ m^4^ mol^–1^ s^–1^ (circles), 10^–6^ m^4^ mol^–1^ s^–1^ (triangles), and 10^–7^ m^4^ mol^–1^ s^–1^ (squares). Results for antiporter parameter values of 10^–8^ m^4^ mol^–1^ s^–1^ are not shown because they were similar for all spatial distributions (in a similar range to the apex only scenarios).

To obtain the optimization results presented in the section *Biophysical Model Optimization*, the plasma membrane Na^+^/H^+^ antiporters were assumed to operate in all root tissue types and developmental zones. The optimization process then determined, to leading order, the most suitable antiporter strengths to match the experimental data, as well as provided a first order demonstration of the location of the Na^+^/H^+^ antiporters. To refine the optimization result in lieu of further experimental data, we consider in this section a manual study of the possible spatial distributions of antiporter activity using the six groups of scenarios listed in the section *Investigating Possible SOS1 Location and Resulting Function*. We predict root Na^+^ contents and Na^+^ fluxes to the shoot under the wide range of transporter strengths (**Figure 5**). The predictions are again compared with experimentally measured values (Davenport et al., 2007) (asterisk symbol and dashed line in **Figure 5**) to identify which spatial distributions give reasonable results. We considered “reasonable” those scenarios that gave rise to outcomes that were within 50% of the wild-type experimental values. These are found within the shaded rectangle in **Figure 5**.

The reasonable model scenarios all have two things in common: plasma membrane antiporters are operating in both the mature xylem parenchyma and at least one tissue type in the mature outer root (see rectangle in **Figure 5**). The reasonable scenarios include plasma membrane Na^+^/H^+^ antiporters operating in: all root tissues, all mature root tissues, the mature cortex and mature xylem parenchyma, or the mature epidermis and mature xylem parenchyma.

Scenarios with plasma membrane antiporters operating in the mature outer root tissues, but not the xylem parenchyma, have low Na^+^ flux to the shoot and relatively low Na^+^ content (purple symbols in **Figure 5**). Plasma membrane antiporters acting in the mature xylem parenchyma, but not the outer root tissues, led to high Na^+^ flux to the shoot and high Na^+^ content (pink symbols in **Figure 5**). Scenarios with plasma membrane antiporters not acting in the mature xylem parenchyma and not in the mature outer root have low Na^+^ flux to the shoot and very high Na root content—even higher than the Na^+^ content measured for the roots of *sos1* mutants (compare brown symbols with dashed line in **Figure 5**).

### SOS1 Functions: Low Root Cytosolic Na^+^

**Figure 6:**
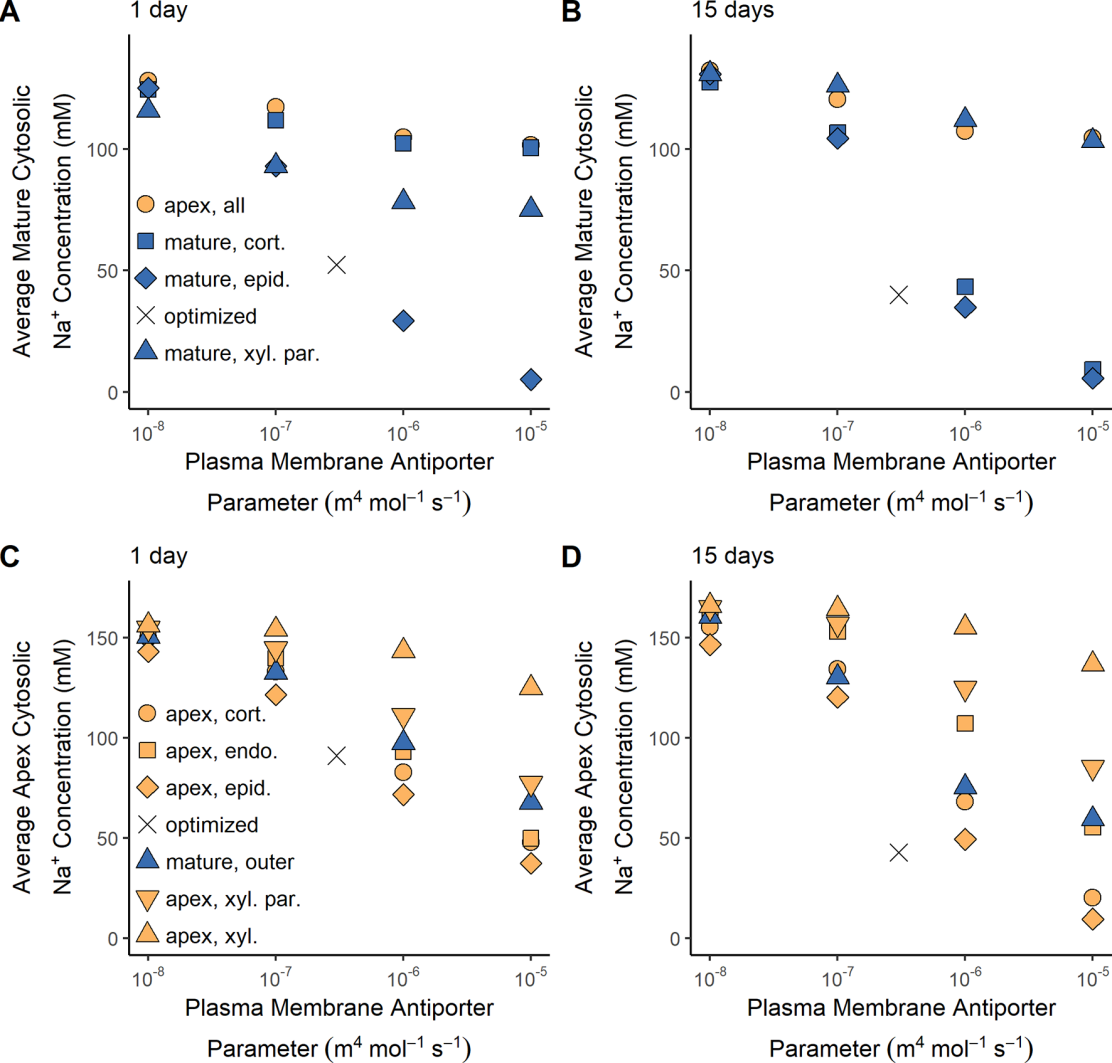
Effect of SOS1 transporter location and strength on cytosolic Na^+^ concentrations in the mature root and apex. **(A)** Average Na^+^ cytosolic concentration in the mature root after 1 day of exposure to NaCl. **(B)** Average Na^+^ cytosolic concentration in the mature root after 15 days of exposure to NaCl. **(C)** Average Na^+^ cytosolic concentration in the root apex after 1 day of exposure to NaCl. **(D)** Average Na^+^ cytosolic concentration in the root apex after 15 days of exposure to NaCl. Plasma membrane spatial distributions in the mature root are shown with blue symbols, while those in the apex are shown with orange symbols. The results obtained using the optimized wild-type antiporter parameters are indicated by crosses. All simulations were conducted with 100 mM NaCl, 7.5 mM KCl, 0.5 mM Ca^2+^, and a pH of 5.7 in the external medium, and all parameters—except the plasma membrane Na^+^/H^+^ antiporter parameters—as shown in **Tables S1** and **S2**.

SOS1 transporters are thought to play a role in controlling root cytosolic Na^+^ concentrations. In this section, we identify how the operation of SOS1 transporters in different root tissues and developmental zones affect the cytosolic Na^+^ concentrations in the mature root (**Figures 6A, B**) and the apex (**Figures 6C, D**). Reasonable cytosolic Na^+^ concentrations (found using the antiporter parameters optimized in the section *Biophysical Model Optimization*) are indicated with crosses (**Figure 6**).

Low root cytosolic Na^+^ concentrations in the mature root in the longer term can be achieved by plasma membrane Na^+^/H^+^ antiporters operating in any of the outer root tissues (**Figure 6B**). However, these antiporters must be operating in the epidermal cells to maintain low levels of cytosolic Na^+^ in the mature root in the short term (**Figure 6A**). This difference in long- and short-term behavior is only evident at high external NaCl concentrations (e.g., it is not present with 50 mM NaCl in the external medium).

Plasma membrane antiporter activity in just the epidermal apical cells achieved the lowest level of cytosolic Na^+^ in the apex, with the effectiveness of the antiporters diminishing in the inner tissues (**Figures 6C, D**). Plasma membrane Na^+^/H^+^ antiporter activity in the outer mature root also lowers the cytosolic Na^+^ concentration in the apex (blue symbols in **Figures 6C, D**). The symplastic connections between the mature root and the apex allow Na^+^ to diffuse between the cytosols of these root regions. As a result, the lower cytosolic Na^+^ concentrations in the mature root due to plasma membrane antiporter activity in the outer mature root drive diffusion of Na^+^ from the apical cell cytosols to the mature root cell cytosols, leading to lower cytosolic Na^+^ concentrations in the apex.

Plasma membrane Na^+^/H^+^ antiporters operating in the mature xylem parenchyma have only a minor effect on cytosolic Na^+^ concentrations in the mature root compared to the effect of these antiporters operating in the outer mature root, especially in the long term (**Figures 6A, B**). This comparatively small effect on cytosolic Na concentrations is a result of the Na^+^ fluxes through the xylem parenchyma antiporters being smaller than those through the antiporters acting on the outer mature cell plasma membranes (e.g., 2.5 times smaller compared to the mature epidermis scenario after 15 days with an antiporter parameter of 10^–5^ m^4^ mol^–1^ s^–1^).

Despite the symplastic connections between the root regions, plasma membrane antiporters operating in any, or all, of the apical cells have a relatively minor effect on the cytosolic Na^+^ concentrations in the mature root (**Figures 6A, B**). Although the antiporters operating in the apex are able to lower the cytosolic Na^+^ concentrations in the cells near the bottom of the mature root, the diffusion of Na^+^ from the mature root cytosols to the apical cell cytosols is not strong enough to substantially lower the Na^+^ concentrations higher up in the mature root. Hence, plasma membrane Na^+^/H^+^ antiporters operating in the root apex are unable to prevent mature root cells higher up in the root from accumulating high levels of cytosolic Na^+^.

To summarize, plasma membrane antiporters must operate in at least some outer root tissues of the mature root in order to maintain reasonably low levels of cytosolic Na^+^ in the mature root, while plasma membrane antiporters operating in the epidermal apical cells are most effective at maintaining low levels of cytosolic Na^+^ in the apex.

### SOS1 Functions: Na^+^ and Water Transport to the Shoot

**Figure 7:**
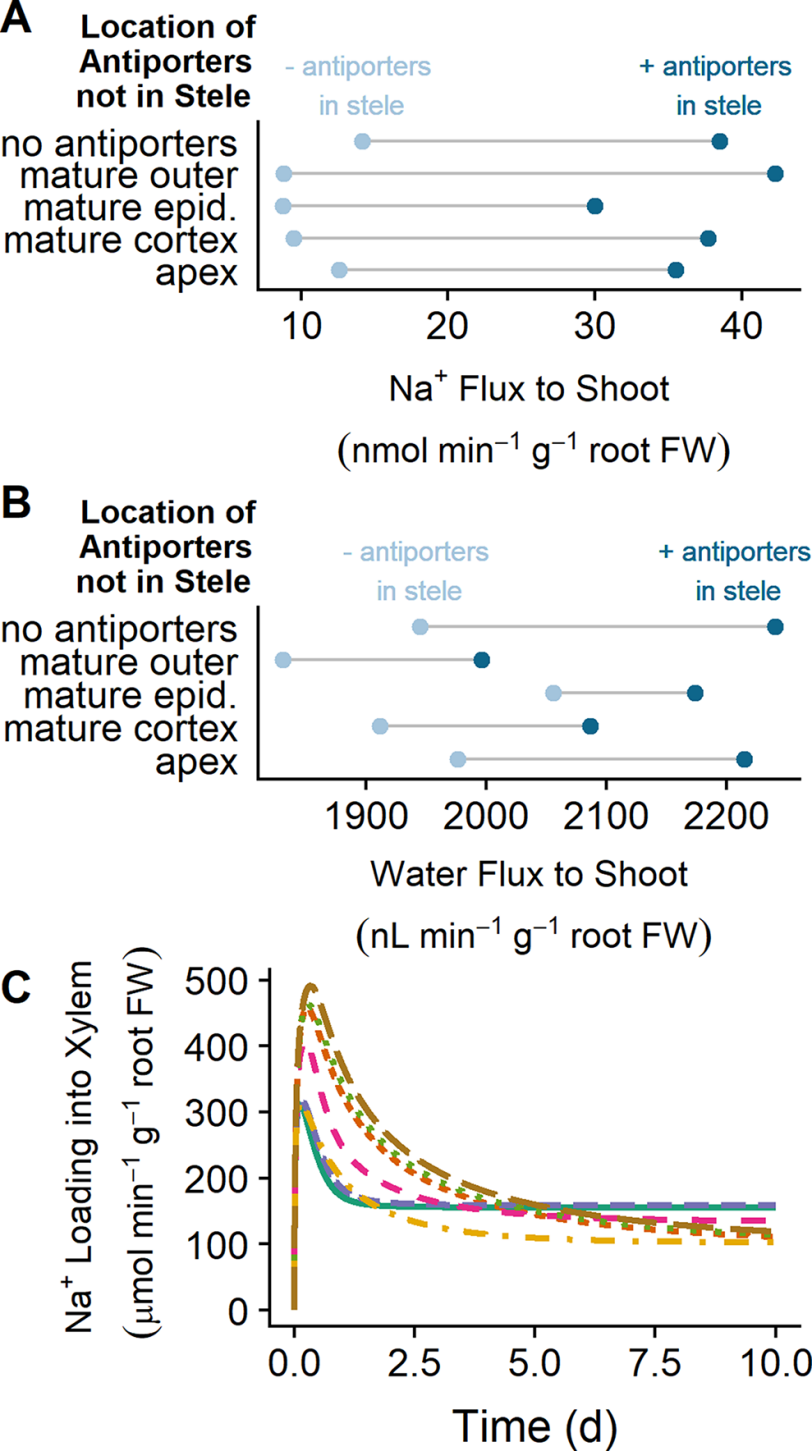
SOS1 transporters operating in the stele load Na^+^ into the xylem, increasing Na^+^ and water transport to the shoot. **(A)** Na^+^ transport to the shoot after 10 days of exposure to NaCl for a range of plasma membrane Na^+^/H^+^ antiporter spatial distributions, showing the effect of having either no plasma membrane antiporters in the mature stele (light blue circles) or plasma membrane antiporters also operating in the mature stele (dark blue circles). **(B)** Water transport to the shoot after 10 days of exposure to NaCl for a range of plasma membrane Na^+^/H^+^ antiporter spatial distributions. **(C)** Na^+^ loading into the xylem *via* plasma membrane Na^+^/H^+^ antiporters over time for a range of spatial distributions of these antiporters, including plasma membrane Na^+^/H^+^ antiporters in: all root tissues (solid green), all mature root tissues (dashed purple), mature epidermis and xylem parenchyma (dot-dashed orange), mature cortex and xylem parenchyma (dashed pink), all apex tissues and mature xylem parenchyma (short-dashed red), apical epidermis and mature xylem parenchyma (dotted green), and mature xylem parenchyma only (long-dashed brown). These fluxes would be negative if antiporters were unloading Na^+^ from the xylem. Simulations were conducted with a plasma membrane antiporter parameter of 10^–7^ m^4^ mol^–1^ s^–1^, and all other parameters as indicated in **Tables S1** and **S2**. Simulations were conducted with 50 mM NaCl, 1.9 mM KCl, 0.5 mM Ca^2+^, and a pH of 5.5 in the external medium

While SOS1 transporters are believed to transport Na^+^ across the plasma membranes of the cells surrounding the xylem, the direction of this transport has been contested (Shi et al., 2002; Munns and Tester, 2008). In this section, we examine how SOS1 transporters influence the flux of Na^+^ to the shoot and identify the direction of Na^+^ transport *via* SOS1 transporters in the stele (**Figure 7**).

Plasma membrane Na^+^/H^+^ antiporters in the mature outer root tissues restrict Na^+^ transport to the shoot, while antiporters in the apex have minimal effect (compare light blue circles in **Figure 7A**). Introducing plasma membrane Na^+^/H^+^ antiporters in the mature stele increases the amount of Na transported to the shoot in all scenarios (compare light and dark blue circles in **Figure 7A**). This leads to an increase in the amount of water transported to the shoot (compare light and dark blue circles in **Figure 7B**).

The xylem parenchyma plasma membrane antiporters are responsible for actively loading Na^+^ into the xylem transpiration stream for all scenarios in which antiporters are operating on the xylem parenchyma plasma membrane (see **Figure 7C**). As a result, the activity of plasma membrane antiporters in the stele leads to the increase in Na flux from the root to the shoot shown in **Figure 7A**.

These results indicate that the role of plasma membrane antiporters in the stele is to actively transport Na^+^ into the transpiration stream, leading to enhanced Na^+^ and water transport from the root to the shoot.

**Figure 8:**
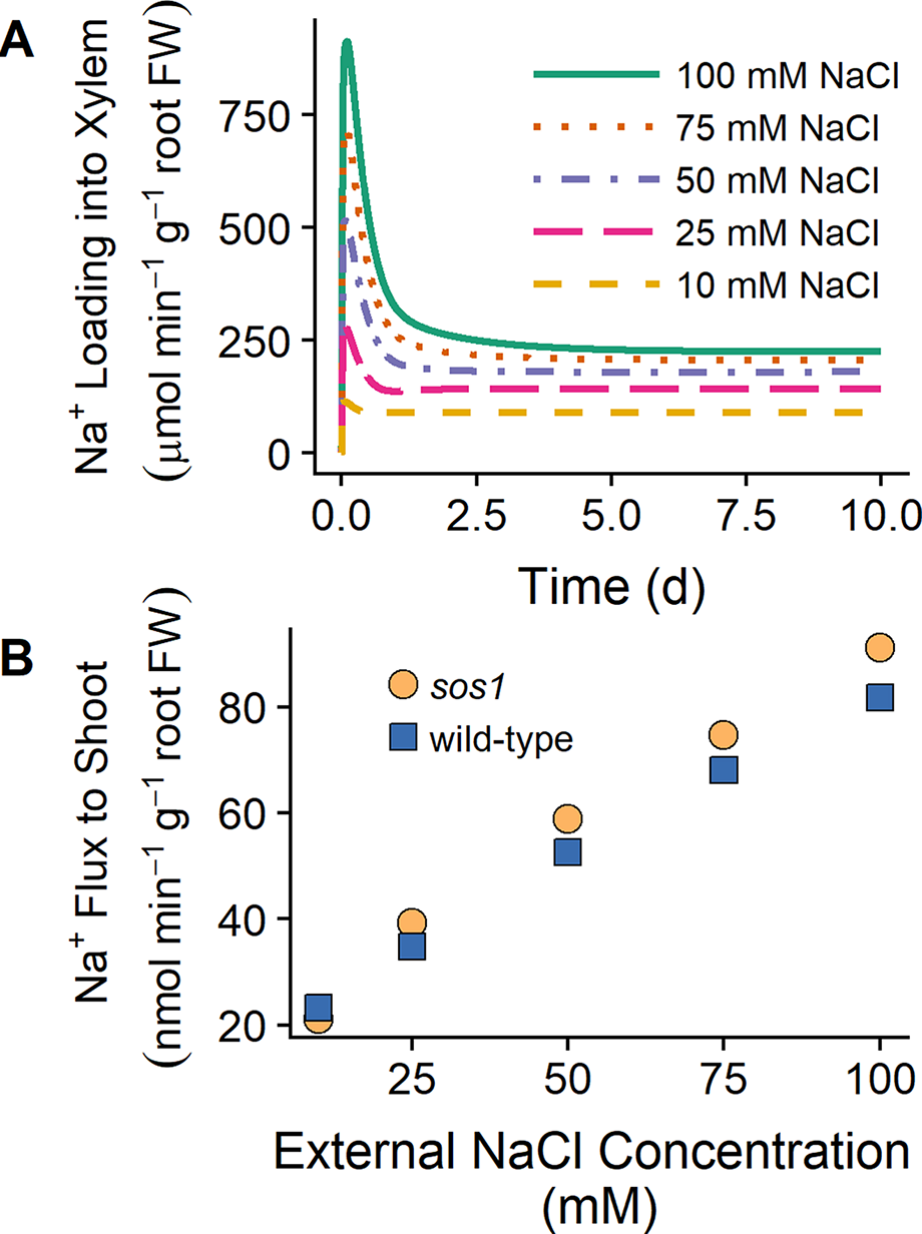
SOS1 transporters load Na^+^ into the xylem for a wide range of external NaCl concentrations. **(A)** Na^+^ loading into the xylem *via* plasma membrane Na^+^/H^+^ antiporters for wild-type scenarios and a range of external NaCl concentrations: 10 mM (dashed orange line), 25 mM (long-dashed pink line), 50 mM (dot-dashed purple line), 75 mM (dotted red line), and 100 mM (solid green line). **(B)** Na^+^ transport to shoot after 1 day of exposure to a range of external NaCl concentrations for *sos1* (orange circles) and wild-type (blue circles) scenarios. Simulations were conducted with parameters as indicated in **Tables S1** and **S2** and with 1 mM KCl, 0.15 mM Ca^2+^, and a pH of 5 in the external medium.

Shi et al.(2002) found that the shoot Na^+^ content was lower for *sos1* mutants compared to wild-type plants at low external NaCl (25 mM), while at high external NaCl (100 mM), the shoot content was higher for *sos1* mutants compared to wild-type plants. One explanation for these observations that Shi et al. (2002) proposed was that SOS1 transporters load Na into the xylem under low external NaCl but unload Na^+^ from the xylem at high external NaCl. However, we found that plasma membrane Na^+^/H^+^ antiporters load Na^+^ into the xylem for external NaCl concentrations ranging from 10 to 100 mM (see **Figure 8A**). **Figure 8B** shows that the model Na^+^ flux to the shoot is lower for *sos1* mutants compared to wild-type plants after 1 day of exposure to low external NaCl (10 mM), while at higher external NaCl, the Na^+^ flux to the shoot is higher for *sos1* mutants compared to wild-type plants. This does not require reversal of the direction of Na transport *via* the plasma membrane antiporters in the stele (see **Figure 8A**).

The results in **Figure 8B** suggest that differences in Na^+^ accumulation in the shoots of wild-type plants and *sos1* mutants would be small in the short term. The differences between the model Na^+^ fluxes to the shoot for these genotypes were also larger after 10 days of exposure to NaCl than after 1 day of exposure (results not shown), suggesting that long-term experiments may be more useful for identifying substantial differences between wild-type plants and *sos1* mutants than short-term experiments.

### Comparison With ^24^Na^+^ Efflux Measurements

**Figure 9:**
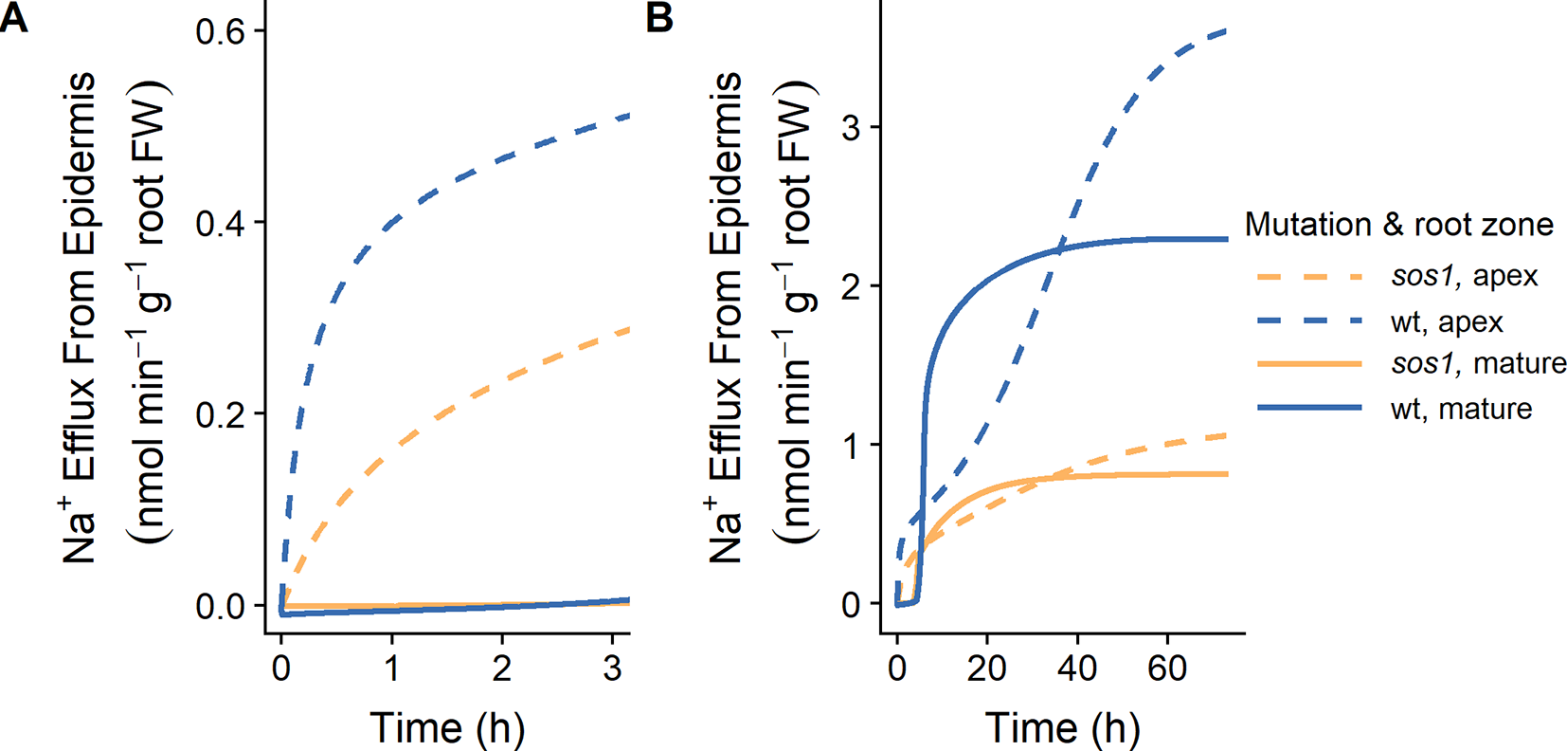
Na^+^ effluxes from root zones differ even if the plasma membrane Na^+^/H^+^ antiporters are operating uniformly across the root zones. **(A)** Initial Na^+^ effluxes after the introduction of 10 mM NaCl. **(B)** Na^+^ effluxes over a longer time frame. Na^+^ effluxes out of the apex (dashed lines) and mature zone (solid lines) are shown for wild-type (wt, blue) and *sos1* (orange) scenarios. Simulations were conducted with parameters as indicated in **Tables S1** and **S2** and with 10 mM NaCl, 1 mM KCl, 0.25 mM Ca^2+^, and a pH of 6 in the external medium. Fluxes are expressed per gram fresh weight (FW) of the relevant root zone.

Our finding that plasma membrane Na^+^/H^+^ antiporters are likely operating in the mature outer root tissues in *Arabidopsis* (see **Figures 5** and **6**) contradicts the conclusions of Hamam et al. (2016) that SOS1 transporters are responsible for significant Na^+^ efflux from the apex, but not the mature root. Their conclusions were based on their measurements of ^24^Na^+^ effluxes: ^24^Na^+^ effluxes from the apices of wild-type *Arabidopsis* roots were approximately three times higher than effluxes from mature root regions. In addition, effluxes from the apex of *sos1* mutants were significantly reduced compared to the wild-type control, while effluxes from the mature root were only slightly reduced (Hamam et al., 2016). However, the total amount of Na^+^ effluxed from a cell is influenced by factors other than just the transporters directly responsible for efflux. For example, **Figure 9** shows that even with identical plasma membrane Na^+^/H^+^ antiporter parameters in the outer mature root and the apex, the Na^+^ effluxes from these two root zones are different, and these fluxes behave differently over time. These differences could be related to other differences between the two root zones, including: the mature root contains functional xylem, and therefore, Na^+^ transport in this region is related to transpiration; the apex does not have active storage of Na^+^ in vacuoles; and there are different transporters and channels operating in the two zones.

With identical plasma membrane Na^+^/H^+^ antiporter parameters in the two root zones, the model results are similar to the fluxes measured by Hamam et al. (2016) up to 3 h after the addition of 10 mM NaCl (**Figure 9A**), and after approximately 40 h of exposure to 10 mM NaCl (**Figure 9B**). For example, the Na^+^ effluxes out of the apex are larger than those out of the mature root, and the effect of the *sos1* knockout is more apparent in the apex.

Comparing the Na^+^ effluxes from different root developmental zones is not straight forward, and the effluxes measured by Hamam et al. (2016) do not necessarily support the conclusion that SOS1 transporters do not contribute significantly to Na^+^ efflux from the outer cells of the mature root.

## Discussion

### SOS1 Transporters Exclude Na^+^ From Root Cytosols and Load Na^+^ Into the Xylem

Our simulations show that SOS1 transporters in the outer mature root exclude Na^+^ from the mature root cytosols and hence the symplast, thus preventing Na^+^ loading to the shoot. The transporters also exclude some Na^+^ from apical root cytosols. SOS1 transporters in the mature stele *load* Na^+^ into the xylem for all the external NaCl concentrations simulated (10 to 100 mM NaCl) and have minimal effect on the root cytosolic Na^+^ concentrations. This active loading of Na^+^ into the xylem counteracts the osmotic stress due to high soil salinity and increases the transport of water to the shoot. SOS1 transporters in the apex, particularly in the epidermal cells, exclude Na^+^ from the apical cytosols but do not significantly exclude Na^+^ from the mature root.

### SOS1 Xylem Loading Versus Unloading of Na^+^

We found no evidence that plasma membrane Na^+^/H^+^ antiporters function to retrieve Na^+^ from the xylem transpiration stream (for any of the external NaCl concentrations simulated). This refutes the active unloading role proposed by Shi et al. (2002), while supporting the thermodynamic analyses of Munns and Tester (2008) and Shabala and Munns (2017). The suggestion that SOS1 transporters load Na^+^ into the xylem under mild salt stress but unload Na^+^ under high salt stress (Shi et al., 2002) was based on the observation that under high salt-stress *sos1* mutants had higher Na^+^ shoot contents relative to wild-type plants, while under mild salt stress, the reverse was true (Shi et al., 2002). However, we have provided evidence that the Na^+^ flux from the root to the shoot can be higher under high salt stress for *sos1* mutants relative to wild-type roots, while under low salt stress, the reverse is possible, without requiring the direction of Na^+^ transport *via* SOS1 to reverse (see **Figure 8**). This observation can be explained by the competing functions of SOS1 transporters in the inner and outer regions of the mature root. The removal of SOS1 function on the plasma membranes of outer root cells would lead to increased Na^+^ transport to the shoot due to increased net uptake of Na^+^ into the root from the external medium, while the removal of SOS1 activity in just the stele would reduce the active loading of Na^+^ into the xylem and hence reduce the flux of Na^+^ to the shoot. As a result, the knockout of *SOS1* in all root tissues could lead to either an increase or decrease in Na^+^ transport to the shoot without requiring the direction of Na^+^ transport through SOS1 transporters in the stele to reverse. This possible explanation of competing functions of SOS1 in different regions of the root was first identified by Shi et al. (2002) as an alternative to their proposal of reversible loading/unloading. However, despite the lack of direct experimental evidence (Shi et al., 2002), the alternative explanation of reversal of the direction of transport *via* SOS1 has been more commonly cited.

Our biophysical model offers an explanation for the counterintuitive use of energy to load Na^+^ into the transpiration stream: *enhanced water transport from the root to the shoot*. Na^+^ loading into the transpiration stream increases the osmotic pressure in the xylem, thus helping the root to maintain water uptake even in the presence of a high osmotic pressure in the external medium due to high soil NaCl concentrations. SOS1 activity in the stele could also lead to an enhancement in water transport in addition to that shown with our model, because transport of Na^+^ to the leaves may allow for energetically efficient osmotic adjustment in the leaves (Munns and Tester, 2008; Shabala and Mackay, 2011; Shabala, 2013; Shabala and Munns, 2017). While this function is important under transpiring conditions, it could be critical when the plant is not transpiring but is still in contact with saline soil as leakage of water out from the root may otherwise result. Active loading of Na^+^ into the transpiration stream could also contribute to slightly lowering the accumulation of Na^+^ in the root.

### Where Are SOS1 Transporters Operating in Roots?

Our model predictions suggest that SOS1 antiporters are operating in: the apex, most likely in the epidermis, at least one tissue in the mature outer root, and in the mature stele. It is instructive to find that predictions of the location of SOS1 transporter activity in the mature outer root tissues does not match the spatial distribution of *SOS1* expression identified by Shi et al. (2002). However, the finding does agree with data available through the *Arabidopsis* eFP browser: Brady et al. (2007) found that *SOS1* expression was highest in root hair cells along the length of the root. On the other hand, these patterns of expression were identified under non-saline conditions. Since *SOS1* mRNA has been found to be significantly up-regulated in roots exposed to salt stress (Shi et al., 2000), it may be more relevant to consider the pattern of *SOS1* expression in the presence of salt stress. In roots exposed to 140 mM NaCl for 1 h, *SOS1* was most highly expressed in the cortex (Dinneny et al., 2008); however, these results did not distinguish between cell types in different developmental zones. Hence, measurements of the spatial distribution of *SOS1* gene expression have led to mixed results. In addition, post-transcriptional and/or post-translational regulations would result in an indirect relationship between indicators of promoter expression and the actual functional location of SOS1 (Shabala et al., 2005). The combined application of a comprehensive biophysical model and physiological experiments may be more beneficial for determining the location of SOS1 transporter activity in roots.

Using model simulations, we have reconciled the conflicting physiological evidence currently available for the location of SOS1 transporter activity. Due to the different transport processes active in different root developmental zones, the available ^24^Na^+^ efflux measurements (Hamam et al., 2016) cannot conclusively demonstrate that SOS1 is inactive in the outer cells of the mature root. We have also shown that reasonable root Na^+^ contents can only be achieved if plasma membrane antiporters are operating in the mature outer root tissues of wild-type plants. This supports the conclusions of Shabala et al. (2005)—based on MIFE H^+^ and K^+^ flux measurements—that SOS1 transporters contribute to transport along the full length of roots.

### Are Other Active Plasma Membrane Na^+^ Transporters Operating in Roots?

As mentioned earlier, to achieve reasonable agreement with experimental measurements of root Na^+^ content using our *sos1* model, a low rather than zero level of plasma membrane Na^+^/H^+^ antiporter activity is required. This suggests either that there is some residual SOS1 activity present in *sos1* mutants, or there is an alternative mechanism responsible for the active Na^+^ efflux from the plasma membranes of outer root cells. Interestingly, plasma membrane vesicles isolated from *sos1* leaves still had some Na^+^/H^+^ antiporter activity, although the source of this activity is unknown (Qiu et al., 2002).

The possibility of an alternative active Na^+^ effluxer has exciting ramifications for improving our understanding of salt transport. This transporter would need to be acting in the outer root tissues in order to significantly affect the root Na^+^ content (see **Figure 5**), and the activity level of this transporter would presumably be lower compared with SOS1 when the latter transporters function normally. *AtNHX8* was a promising candidate for this active efflux role as it is expressed in root cells and has a similar structure to *SOS1*. However, An et al. (2007) demonstrated that AtNHX8 is a Li^+^/H^+^ plasma membrane antiporter that does not transport Na^+^. It is possible that a member of the CHX family of transporters could instead fulfill this role. To date, only AtCHX21 has been identified to transport Na^+^. However, AtCHX21 has been found to operate on the plasma membrane of endodermal cells transporting Na^+^ into the stele (Hall et al., 2006). To date, no other active Na^+^ effluxer has been confirmed to operate on the plasma membranes of outer root cells (Plett and Møller, 2010; Shabala and Munns, 2017).

### Implications for Achieving Enhanced Salt Tolerance

Based on the various locations and corresponding functional outcomes of the SOS1 transporter scenarios that we have studied, it seems highly likely that overexpressing *SOS1* in only the outer root tissues would lead to lower root cytosolic Na^+^ concentrations, potentially resulting in improved salt tolerance. In addition, overexpressing *SOS1* in the outer root cells and knockout or downregulation of *SOS1* in just the stele would lead to reduced Na^+^ flux to the shoot. Given that a reduced Na^+^ flux to the shoot following the overexpression of *AtHKT1;1* in the stele led to an improvement in salt tolerance (Møller et al., 2009), it is likely that the former tissue-specific changes to the expression of *SOS1* would similarly lead to improved salt tolerance. However, we have also shown that Na^+^ transport to the shoot is important in order for the plant to maintain water uptake under salt stress. Therefore, the combination of changes in *SOS1* expression suggested above could potentially result in such low levels of Na^+^ transport to the shoot that the plant’s reduced ability to take up water would negate any benefits in reduced Na^+^ toxicity in the leaf. Worse still, there could be a loss of water due to a negative electrochemical water potential under non-transpiring conditions (see Arsova et al. (2019)). Our results highlight the importance of considering the tradeoff between Na^+^ exclusion and water uptake.

### Comments on Experimental Comparisons

It is to be expected that any realistic biophysical model will occupy a position in a large parameter space due to the large number of transporters and channels that contribute to the salt-stress response of roots. It follows that, for a model to predict meaningful outcomes, the parameters governing the transport mechanisms that feature must reflect those of actual transport processes occurring in real roots. We have identified reasonable parameter values by comparing the predictions of our comprehensive biophysical model with many experiments, measuring a wide range of variables (including Na^+^ and K^+^ root content; xylem concentrations of Na^+^, K^+^, and anions; and epidermal membrane potentials) for both wild-type *Arabidopsis* and *sos1* mutant roots.

Although our comprehensive biophysical model is an approximation to the complex transport system operating in real roots, the optimized model predictions compared well with experimental data found under a wide range of conditions. On the other hand, the sizable difference between the optimized model results and the experimental data that was found with the anion concentration in the xylem of a salt-stressed root deserves some discussion. At present, Cl^–^ is the only mobile anion in our model, and therefore, this nominal “Cl^–^ ion” plays the role of all free anions present in an actual plant root. Consequently, the concentrations predicted for this sole anion were as required to satisfy the constraint of electroneutrality. In a physical root, all anions contribute to this requirement (inversely as their valency). However, our anion concentrations in **Figure 4** are compared here with the explicitly measured concentrations of only two monovalent anions (Cl^–^ and $NO_{3}^{-}$). Adding $NO_{3}^{-}$ transport may enable the model to better capture the differences between the anion concentrations in the unstressed and salt-stressed scenarios. Nevertheless, the model predictions suggest that selective experimental determination of specific anions may overlook other neutralizing ion contributions. What is clear, however, is that the discrepancy between the model and experimental anion xylem concentrations does not substantially affect the model results or conclusions regarding the location and function of SOS1 transporters.

Unfortunately, not all of the available data for *sos1* mutants was useful for the optimization process. The only xylem ion concentrations currently available for *sos1* mutants were measured using excised roots (Shi et al., 2002). Interestingly, using our model based on parameters for a transpiring root but applied to an excised root (modeled by removing transpiration), we were unable to obtain Na^+^ xylem concentrations comparable to those measured by Shi et al. (2002) for *sos1* mutants (see **Figure S3**). However, it is likely that transport functions for excised roots differ from roots of intact, transpiring plants. The processes driving transport into the xylem in excised roots are poorly understood (Wegner, 2017), and this is an area for future investigation using our model.

Additional experimental measurements, such as measurements of cytosolic Na^+^ concentrations in different *Arabidopsis* root tissues and ion concentrations in the root xylem of intact *sos1* mutant plants could help to further verify our model predictions and shed additional light on the functions of SOS1 transporters.

### Future Work

Our focus here has been on modeling ion and water transport in *Arabidopsis* plants, due to the availability of suitable experimental data. Consequently, the parameter values that we have identified are tuned to *Arabidopsis* and may not be transferrable to other species. However, our comprehensive biophysical model can be easily adapted to represent species other than *Arabidopsis*, by altering the number of model tissue layers, the model root geometry, and inclusion of other processes as required (see, for example, Munns et al. (2019a) and Arsova et al. (2019)). It is therefore possible, as more experimental data becomes available, to investigate whether the outcomes predicted here are germane to only *Arabidopsis* or have wider applicability.

SOS1 regulation is another interesting aspect of SOS1 function that could be investigated in the future using our model. For example, SOS1 activity has been shown to be up-regulated by the protein kinase complex SOS2–SOS3 (Qiu et al., 2002; Quintero et al., 2002, Quintero et al., 2011). In this study, we did not explicitly model regulation of SOS1 transporter activity as the current model (without the added complexity of regulation) was a necessary first step toward understanding the role of SOS1 transporters. The availability of additional experimental data in the future would increase the feasibility of including this additional level of detail in the model, allowing the effects of the regulation of SOS1 activity to be explored in detail. However, it is possible to interpret our existing model results in light of SOS1 regulation. For example, different model antiporter transport parameter values (see **Figure 5**) can be viewed as representing different levels of SOS1 activation.

Our model includes transport of ions through numerous, interacting, transporters and channels (see **Figure 2**). One interaction that could be of particular interest for future model investigations is the unloading of Na^+^ from the xylem transpiration stream by HKT1;1 (Møller et al., 2009), which opposes the loading of Na^+^ into the xylem transpiration stream by SOS1 transporters, introducing the possibility of futile Na^+^ cycling across the xylem parenchyma plasma membranes. Transport of Na^+^ through HKT1;1 is already included in our model (see the section *Model Description*), so the model could be used in the future to explore in detail how these transporters interact.

## Conclusions

Based on our model simulations and new interpretations of existing experimental data, SOS1 transporters are likely to be operating in the stele and at least one tissue type in the outer mature root of *Arabidopsis*, as well as in at least the epidermal cells of the apex. We have shown that SOS1 transporters in the outer cells of the mature root restrict the level of Na^+^ in root cell cytosols and limit the flux of Na^+^ to the shoot. In contrast, SOS1 transporters in the mature stele actively load Na^+^ into the xylem, enhancing the flux of both Na^+^ and water to the shoot. In the root apex, it is sufficient for SOS1 transporters to operate in the epidermis to prevent the toxic build-up of Na^+^ in apical cell cytosols.

We have demonstrated that mathematical modeling is a crucial tool for understanding the complex, interacting, transport processes contributing to salt tolerance.

## Data Availability Statement

All datasets generated for this study are included in the manuscript and the **Supplementary Files**.

## Author Contributions

KF and SM contributed equally to the research design, the interpretation of results and the writing of the manuscript. KF performed the simulations and produced the figures.

## Funding

The authors would also like to acknowledge financial support from the South Australian Department of State Development (Grant Nr: IRGP 22).

## Conflict of Interest

The authors declare that the research was conducted in the absence of any commercial or financial relationships that could be construed as a potential conflict of interest.

## Footnotes

1 Data available using the Arabidopsis eFP Browser, http://bar.utoronto.ca/efp/cgi-bin/efpWeb.cgi.
