# Supplementary material for: A Comprehensive Biophysical Model of Ion and Water Transport in Plant Roots. II. Clarifying the Roles of SOS1 in the Salt-Stress Response in Arabidopsis
Source: Front Plant Sci. 2019 Sep 18;10:1121. doi: 10.3389/fpls.2019.01121 (PMC6759596; doi:10.3389/fpls.2019.01121)
Supplement: Supplementary file 1 [file DataSheet_1.pdf]

Supplementary Material

S.1. INTRODUCTION

This Supplementary Material summarizes our model equations and solution method. Additional details, including justifications of model assumptions, are available in the Supplementary Material for Foster and Miklavcic (2017) and the Supplementary Data for Foster and Miklavcic (2015).

The model root cylinder is discretized in the axial and radial directions to create annular cylinders that are the height and width of a single cell. Each of these annular cylinders (referred to as an 'element') is designated by a discrete coordinate (α, j), where: α refers to the radial tissue type ($\alpha = 1$ for the epidermis, $\alpha = 2$ for the cortex, $\alpha = 3$ for the endodermis, $\alpha = 4$ for the xylem parenchyma and $\alpha = 5$ for the xylem); and j refers to the distance from the root tip in cell layers (there are a total of 30 cell layers in the axial direction). Each root element contains multiple cells, with: the epidermis containing 19 cells; the cortex containing 8 cells; the endodermis containing 8 cells; the xylem parenchyma containing 12 cells; and the immature xylem containing 2 cells (Dolan et al., 1993).

S.2. WATER TRANSPORT

Assuming that the solution density is constant, each tissue is full of liquid, the cells are fixed in size and the vacuole volume is variable, the mass-balance equations for the apoplastic, cytosolic and vacuolar fluid simplify to:

$$Q^{a,rad}_{in,\alpha,j} - Q^{a,rad}_{out,\alpha,j} + Q^{a,ax}_{in,\alpha,j} - Q^{a,ax}_{out,\alpha,j} + N^{cells}_{\alpha,j} Q^{p}_{\alpha,j} = 0, \tag{S1}$$

$$Q^{s,rad}_{in,\alpha,j} - Q^{s,rad}_{out,\alpha,j} + Q^{s,ax}_{in,\alpha,j} - Q^{s,ax}_{out,\alpha,j} - N^{cells}_{\alpha,j} Q^{p}_{\alpha,j} = 0, \tag{S2}$$

$$\frac{dV^{v}_{\alpha,j}}{dt} = Q^{t}_{\alpha,j}. \tag{S3}$$

Q^a terms refer to apoplastic water flow rates, Q^s terms refer to symplastic water flow rates, Q^p is the water flow rate across the plasma membrane of each cell (positive into the apoplast), Q^t is the water flow rate across the tonoplast of each cell (positive into the vacuole), $N^{cells}_{\alpha,j}$ is the number of cells in element (α, j) and $V^{v}_{\alpha,j}$ is the volume of the vacuole of each cell in each (α, j) element. Superscripts (rad/ax) indicate whether the apoplastic and symplastic flows are radial or axial, while subscripts (in/out) indicate whether these flows are directed into or out of (α, j).

The water flow rates are simulated using nonequilibrium thermodynamics (Foster and Miklavcic, 2017):

$$Q^{a,rad}_{in,\alpha,j} = A^{a,rad}_{\alpha,j} L^{a,rad}_{p:\alpha,j} \left(p^{a}_{\alpha-1,j} - p^{a}_{\alpha,j}\right), \tag{S4}$$

$$Q^{a,ax}_{in,\alpha,j} = L^{a,ax}_{p:\alpha,j-1} A^{a,ax}_{\alpha,j-1} \left(p^{a}_{\alpha,j-1} - p^{a}_{\alpha,j} - \rho_w g l^{ax}_{\alpha,j}\right), \tag{S5}$$

$$Q^{s,rad}_{in,\alpha,j} = A^{s,rad}_{\alpha,j} L^{s}_{p} \left[p^{c}_{\alpha-1,j} - p^{c}_{\alpha,j} - R_g T \sum_{n=1}^{N^{c}_{osm}} \sigma^{s,n} \left(C^{c,n}_{\alpha-1,j} - C^{c,n}_{\alpha,j} \right) \right], \tag{S6}$$

$$Q^{s,ax}_{in,\alpha,j} = A^{s,ax}_{\alpha,j-1} L^{s}_{p} \left[p^{c}_{\alpha,j-1} - p^{c}_{\alpha,j} - \rho_w g l^{ax}_{\alpha,j} - R_g T \sum_{n=1}^{N^{c}_{osm}} \sigma^{s,n} \left(C^{c,n}_{\alpha,j-1} - C^{c,n}_{\alpha,j} \right) \right], \quad \text{(S7)}$$

$$Q^{p}_{\alpha,j} = A^{p}_{\alpha,j} L^{p}_{p} \left[p^{c}_{\alpha,j} - p^{a}_{\alpha,j} - R_g T \left(\sum_{n=1}^{N^{c}_{osm}} C^{c,n}_{\alpha,j} - \sum_{n=1}^{N^{a}_{osm}} C^{a,n}_{\alpha,j} \right) \right], \quad \text{(S8)}$$

$$Q^{t}_{\alpha,j} = A^{t}_{\alpha,j} L^{t}_{p} R_g T \left(- \sum_{n=1}^{N^{c}_{osm}} C^{c,n}_{\alpha,j} + \sum_{n=1}^{N^{v}_{osm}} C^{v,n}_{\alpha,j} \right). \quad \text{(S9)}$$

$A^{a/s/p/t}_{\alpha,j}$ is the area through which the apoplastic/symplastic/trans-plasma membrane/trans-tonoplast water transport occurs and $L_{p:\alpha,j}$ is the relevant water permeability. $C^{a/c/v,n}_{\alpha,j}$ is the concentration of ion n in the apoplast/cytosol/vacuole in element (α, j), R_g is the Universal gas constant (8.314 J mol^{-1} K^{-1}), T is the temperature and N_{osm} is the total number of solute species contributing to the osmotic pressure (this varies between cellular compartments). $p^{a}_{\alpha,j}$ is the hydrostatic pressure in the apoplast, $p^{c}_{\alpha,j}$ is the cell turgor pressure, $\sigma^{s,n}$ is the solute reflection coefficient for transport via plasmodesmata, ρ_w is the density of water, g is the acceleration due to gravity and $l^{ax}_{\alpha,j}$ is the height of element (α, j).

The radial apoplastic/symplastic water flow rate out of element (α, j) is equal to the radial apoplastic/symplastic inflow into its neighbor element. Similarly, the axial apoplastic/symplastic water flow rate out of element (α, j) is equal to the axial apoplastic/symplastic inflow into its neighbor element.

S.3. ION TRANSPORT

The mole balance equations for mobile ions, n (n = Na^{+}, Cl^{-}, K^{+}, H^{+}), in the apoplast, cytosol and vacuole of each (α, j) element are given by,

$$\frac{d}{dt}\left(V^{a}_{\alpha,j} C^{a,n}_{\alpha,j} \right) = S^{a,rad,n}_{in,\alpha,j} - S^{a,rad,n}_{out,\alpha,j} + S^{a,ax,n}_{in,\alpha,j} - S^{a,ax,n}_{out,\alpha,j} + N^{cells}_{\alpha,j} S^{p,n}_{\alpha,j} + R^{bind,n}_{\alpha,j}, \quad \text{(S10)}$$

$$\frac{d}{dt}\left(V^{c}_{\alpha,j} C^{c,n}_{\alpha,j} \right) = \frac{1}{N^{cells}_{\alpha,j}} \left(S^{s,rad,n}_{in,\alpha,j} - S^{s,rad,n}_{out,\alpha,j} + S^{s,ax,n}_{in,\alpha,j} - S^{s,ax,n}_{out,\alpha,j} \right) - S^{p,n}_{\alpha,j} - S^{t,n}_{\alpha,j}, \quad \text{(S11)}$$

$$\frac{d}{dt}\left(V^{v}_{\alpha,j} C^{v,n}_{\alpha,j} \right) = S^{t,n}_{\alpha,j}. \quad \text{(S12)}$$

$V^{a/c/v}_{\alpha,j}$ is the volume of the apoplast/cytosol/vacuole in (α, j). S^{n} terms refer to fluxes of solute n, with superscripts $a/s/p/t$ indicating apoplastic/symplastic/trans-plasma membrane/trans-tonoplast fluxes. Superscripts rad/ax indicate whether the apoplastic and symplastic flows are radial or axial, while subscripts in/out indicate whether the flows are directed into or out of (α, j). $R^{bind,n}_{\alpha,j}$ is the net rate of ion dissociation from the binding reaction with fixed negative groups that are present in the apoplast. The mole balance equation for cations bound to fixed

negative ions in the apoplastic compartment of each (α, j) element is given by,

$$\frac{d\left(V^a_{\alpha,j}C^{a,nB}_{\alpha,j}\right)}{dt} = -R^{bind,n}_{\alpha,j}, \tag{S13}$$

where $C^{a,nB}_{\alpha,j}$ refers to the concentration of bound cations (HB, NaB, KB).

The trans-plasma membrane fluxes are given by,

$$S^{p,n}_{\alpha,j} = \begin{cases} J^{p,H}_{A:\alpha,j} + J^{p,H}_{XNa:\alpha,j} + J^{p,H}_{SK:\alpha,j} + J^{p,H}_{SCl:\alpha,j} & \text{if } n = \text{H}^+, \\ J^{p,Na}_{C:\alpha,j} + J^{p,Na}_{XNa:\alpha,j} & \text{if } n = \text{Na}^+, \\ J^{p,K}_{C:\alpha,j} + J^{p,K}_{ORC:\alpha,j} + J^{p,K}_{IRC:\alpha,j} + J^{p,K}_{SK:\alpha,j} & \text{if } n = \text{K}^+, \\ J^{p,Cl}_{C:\alpha,j} + J^{p,Cl}_{SCl:\alpha,j} & \text{if } n = \text{Cl}^-, \end{cases} \tag{S14}$$

and the trans-tonoplast fluxes are given by,

$$S^{t,n}_{\alpha,j} = \begin{cases} J^{t,H}_{A:\alpha,j} + J^{t,H}_{XNa:\alpha,j} + J^{t,H}_{XK:\alpha,j} & \text{if } n = H^+, \\ J^{t,Na}_{C:\alpha,j} + J^{t,Na}_{XNa:\alpha,j} & \text{if } n = Na^+, \\ J^{t,K}_{C:\alpha,j} + J^{t,K}_{XK:\alpha,j} & \text{if } n = K^+, \\ J^{t,Cl}_{C:\alpha,j} & \text{if } n = Cl^-. \end{cases} \tag{S15}$$

J terms represent the trans-membrane ion fluxes through each type of membrane transport protein (fluxes are positive out of the cytosol). Superscripts denote the relevant membrane (p for plasma membrane and t for tonoplast) and ion (H^+, Na^+, K^+, Cl^-). Subscripts refer to the type of transport protein carrying the flux: A for H^+ pumps, XNa for Na^+/H^+ antiporters, XK for K^+/H^+ antiporters, SK for K^+/H^+ symporters, SCl for Cl^-/$2H^+$ symporters, C for voltage insensitive channels, ORC for K^+ outward rectifying channels (ORCs) and IRC for K^+ inward rectifying channels (IRCs).

S.3.1 Apoplastic and symplastic ion transport

The fluxes of ions through the apoplast and symplast are represented by an extended Nernst-Planck equation (van der Horst et al., 1995),

$$\begin{aligned} S^{a/s,rad,n}_{in,\alpha,j} &= -k^{a/s,rad,n}_{\alpha,j}A^{a/s,rad}_{\alpha,j}\left[C^{a/c,n}_{\alpha,j} - C^{a/c,n}_{\alpha-1,j} + \frac{Z^nC^{a/c,n}_{\alpha-1,j}F}{R_gT}\left(\psi^{a/c}_{\alpha,j} - \psi^{a/c}_{\alpha-1,j}\right)\right] \\ &+ \left(1-\sigma^{a/s,n}\right)C^{a/c,n}_{\alpha-1,j}Q^{a/s,rad}_{in,\alpha,j}, \end{aligned} \tag{S16}$$

$$\begin{aligned} S^{a/s,ax,n}_{in,\alpha,j} &= -k^{a/s,ax,n}_{\alpha,j-1}A^{a/s,ax}_{\alpha,j-1}\left[C^{a/c,n}_{\alpha,j} - C^{a/c,n}_{\alpha,j-1} + \frac{Z^nC^{a/c,n}_{\alpha,j-1}F}{R_gT}\left(\psi^{a/c}_{\alpha,j} - \psi^{a/c}_{\alpha,j-1}\right)\right] \\ &+ \left(1-\sigma^{a/s,n}\right)C^{a/c,n}_{\alpha,j-1}Q^{a/s,ax}_{in,\alpha,j}. \end{aligned} \tag{S17}$$

$k^{a/s,rad,n}$ ($k^{a/s,ax,n}$) is the radial (axial) diffusive permeability of ion n in the apoplast/symplast, Z^n is the valence of ion n, $\psi^{a/c}$ is the electric potential in the apoplast/cytosol and F is Faraday's constant (96 485 C mol^{-1}). The solute reflection coefficient in the apoplast ($\sigma^{a,n}$) is assumed to be zero.

The radial outflow of ion n from the apoplast (symplast) of an element is equal to the radial apoplastic (symplastic) inflow into its neighboring element. Similarly, the axial outflow of ion n from the apoplast (symplast) of an element is equal to the axial apoplastic (symplastic) inflow into its neighbor.

The ion permeabilities for each ion, n, were calculated using (Foster and Miklavcic, 2017),

$$k^{a,rad,n} = \frac{D_e^{a,n}}{d_\alpha}, \tag{S18}$$

$$k^{a,ax,n} = \frac{D_e^{a,n}}{l_j^{ax}}, \tag{S19}$$

$$k^{s,rad/ax,n} = \frac{D_e^{s,n}}{t_{cw}}, \tag{S20}$$

where $D_e^{a/s,n}$ is the effective diffusion coefficient of ion n in the apoplast/symplast, d_α is the thickness of each tissue region and t_{cw} is the cell wall thickness (*i.e.*, the length of the plasmodesmata). $D_e^{a,n}$ is assumed to equal $\frac{D^n}{5}$ (Foster and Miklavcic, 2017), where D is the bulk diffusion coefficient of ion n. The effective symplastic diffusion coefficient for each ion n was determined using $D_e^{s,n} = H^n D^n$ where H^n is the hindrance factor for diffusion of solute n through a cylindrical pore (Liesche and Schulz, 2013; Dechadilok and Deen, 2006), given by,

$$\begin{aligned} H^n = 1 &+ \frac{9}{8}\gamma_n \ln \gamma_n - 1.56034\gamma_n + 0.528155\gamma_n^2 + 1.91521\gamma_n^3 - 2.81903\gamma_n^4 + 0.270788\gamma_n^5 \\ &+ 1.10115\gamma_n^6 - 0.435933\gamma_n^7, \end{aligned} \tag{S21}$$

where γ_n is the solute radius divided by the pore radius. The resulting effective diffusion coefficients for ions in the symplast and apoplast are summarized in Table S2.

The solute reflection coefficients for transport via the plasmodesmata were calculated using $\sigma^{s,n} = 1 - W_n$ (Liesche and Schulz, 2013), where W is the hindrance factor for convection through a cylindrical pore (Dechadilok and Deen, 2006), given by,

$$W_n = (1-\gamma_n)\left(\frac{1 + 3.867\gamma_n - 1.907\gamma_n^2 - 0.834\gamma_n^3}{1 + 1.867\gamma_n - 0.741\gamma_n^2}\right). \tag{S22}$$

The resulting solute reflection coefficients are summarized in Table S2.

S.3.2 Passive transmembrane transport

Passive Na^+ transport is assumed to occur through voltage insensitive channels (VICs), while passive K^+ transport is assumed to occur through VICs, inward rectifying channels (IRCs) and outward rectifying channels (ORCs).

The passive flux of ion n ($n = \text{Na}^+$, K^+ and Cl^-) through voltage insensitive channels (VICs) is modeled using the Goldman-Hodgkin-Katz (GHK) current equation (Keener and Sneyd, 2009),

$$J^{p/t,n}_{C:\alpha,j} = \frac{A^{i,p/t}_{\alpha,j} P^{p/t,n}_{C:\alpha,j} Z^n F \Delta\psi^{p/t}_{\alpha,j}}{R_g T} \left[\frac{C^{c,n}_{\alpha,j} - C^{a/v,n}_{\alpha,j} \exp\left(\frac{-Z^n \Delta\psi^{p/t}_{\alpha,j} F}{R_g T} \right)}{1 - \exp\left(\frac{-Z^n \Delta\psi^{p/t}_{\alpha,j} F}{R_g T} \right)} \right], \tag{S23}$$

where $P^{p/t,n}_{C:\alpha,j}$ is the permeability of the plasma membrane/tonoplast channel for ion n expressed per unit area of membrane, $A^{i,p/t}$ is the initial area of the plasma/tonoplast membrane, and $\Delta\psi^{p/t}_{\alpha,j} = \psi^c - \psi^{a/v}$ is the plasma membrane/tonoplast potential difference.

Na^+ and K^+ are assumed to be transported through plasma membrane nonselective cation channels (NSCCs) in the outer root tissues. The permeability of these channels is dependent on the external Ca^{2+} concentration (Demidchik and Tester, 2002). This dependence is simulated by multiplying the maximal NSCC permeability by the following factor (Hills et al., 2012),

$$P^p_{NSCC:\alpha,j} = 1 - \frac{\left(C^{Ca}\right)^h}{\left(C^{Ca}\right)^h + \left(K_{NSCC}\right)^h}, \tag{S24}$$

where C^{Ca} is the concentration of Ca^{2+} in the external medium, h is a Hill coefficient and K_{NSCC} is a binding constant.

Xue et al. (2011) demonstrated, using patch-clamp studies, that Na^+ transport via HKT1;1 in *Arabidopsis* stelar cells is passive with no strong voltage dependence. As a result, we used Eq. (S23) to model Na^+ transport across the xylem parenchyma plasma membranes via AtHKT1;1.

We modeled K^+ fluxes through voltage dependent channels using the GHK current equation (Eq. (S23)), with the addition of a term representing the voltage dependence of the channel open probability,

$$J^{p,K}_{IRC/ORC:\alpha,j} = \frac{A^{i,p}_{\alpha,j} P^o_{IRC/ORC:\alpha,j} P_{IRC/ORC:\alpha,j} F \Delta\psi^p_{\alpha,j}}{R_g T} \left[\frac{C^{c,K}_{\alpha,j} - C^{a,K}_{\alpha,j} \exp\left(\frac{-\Delta\psi^p_{\alpha,j} F}{R_g T} \right)}{1 - \exp\left(\frac{-\Delta\psi^p_{\alpha,j} F}{R_g T} \right)} \right], \tag{S25}$$

where $P^o_{IRC/ORC}$ is the IRC/ORC open probability and $P_{IRC/ORC}$ is the maximal IRC/ORC permeability. Assuming two state channels (open/closed), the open probabilities are given by

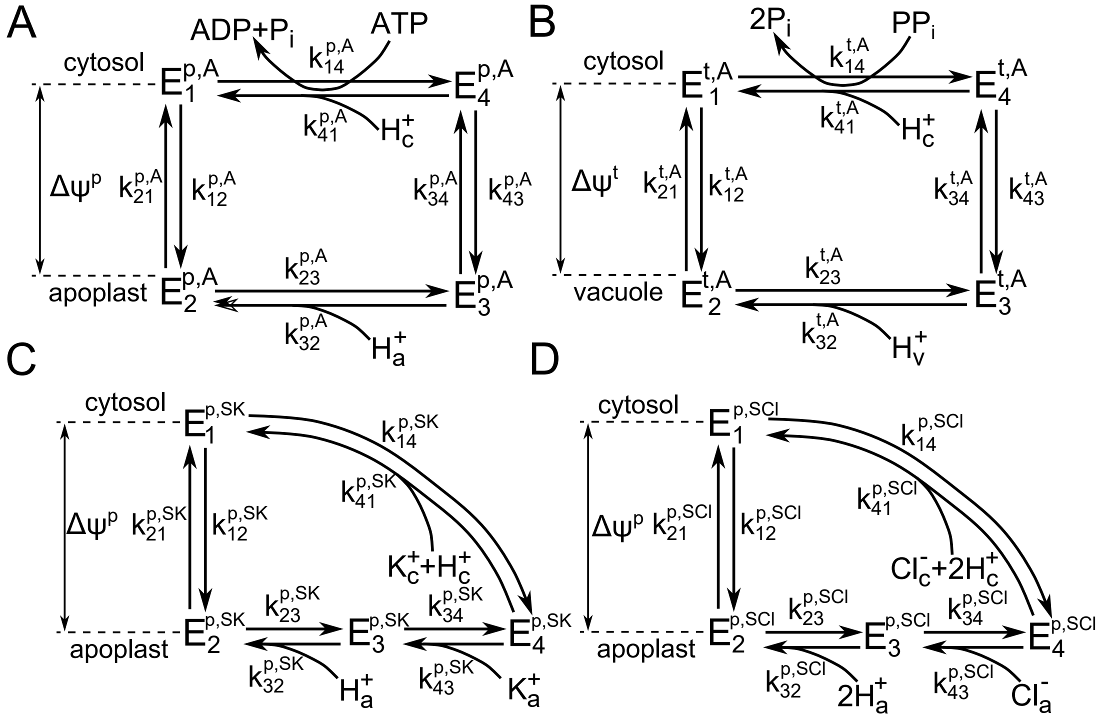

Figure S1. The four state carrier cycles used to model pumps and symporters. Carrier cycles are shown for **(A)** the plasma membrane H$^+$ pump, **(B)** the tonoplast H$^+$ pump, **(C)** the K$^+$/H$^+$ plasma membrane symporter, and **(D)** the Cl$^-$/2H$^+$ plasma membrane symporter. $\psi^{p/t}$ refers to the trans-plasma membrane/trans-tonoplast membrane potentials, E's refer to carrier states, while k's refer to rate constants. Adapted from Foster and Miklavcic (2017).

(Chowdhury and Chanda, 2012),

$$P^o_{IRC:\alpha,j} = \frac{1}{1 + \exp\left[\frac{FZ_g^{IRC}}{R_gT}\left(\Delta\psi^p_{\alpha,j} - \Delta\psi_{50}^{IRC}\right)\right]}, \tag{S26}$$

$$P^o_{ORC:\alpha,j} = \frac{1}{1 + \exp\left[-\frac{FZ_g^{ORC}}{R_gT}\left(\Delta\psi^p_{\alpha,j} - \Delta\psi_{50:\alpha,j}^{ORC}\right)\right]}, \tag{S27}$$

where $Z_g^{IRC/ORC}$ is the IRC/ORC gating charge and $\Delta\psi_{50}^{IRC/ORC}$ is the trans-plasma membrane potential at which half the inward/outward rectifying channels are open.

The voltage-dependent gating of K$^+$ ORCs is affected by the external K$^+$ concentration, with increasing apoplastic K$^+$ shifting the range over which the channel is active to more positive potentials (Ivashikina et al., 2001; Johansson et al., 2006). To simulate this behavior, $\Delta\psi_{50}^{ORC}$ is given by (Hills et al., 2012),

$$\Delta\psi_{50:\alpha,j}^{ORC} = \frac{R_gT}{F} ln \frac{C^{a,K}_{\alpha,j}}{K^{50,a}_{ORC}}, \tag{S28}$$

where $K^{50,a}_{ORC}$ is the apoplastic K$^+$ concentration at which $\Delta\psi_{50}^{ORC} = 0$.

S.3.3 Active transmembrane transport via H^+ pumps

The plasma membrane and tonoplast H^+ pumps are modeled by four state carrier cycles (Hills et al., 2012) as shown in Figures S1A and S1B. H^+ fluxes through the plasma membrane/tonoplast pumps are given by,

$$J^{p/t,H}_{A:\alpha,j} = \frac{N^{p/t,H}_{A:\alpha,j} A^{i,p/t}_{\alpha,j}}{N_A D^{p/t,H}_{A:\alpha,j}} \left(k^{p/t,A}_{12:\alpha,j} k^{p/t,A}_{23} k^{p/t,A}_{34} k^{p/t,A}_{41:\alpha,j} - k^{p/t,A}_{14} k^{p/t,A}_{21:\alpha,j} k^{p/t,A}_{32:\alpha,j} k^{p/t,A}_{43} \right), \quad \text{(S29)}$$

where the denominator term is given by,

$$\begin{aligned} D^{p/t,H}_{A:\alpha,j} = {} & k^{p/t,A}_{14} k^{p/t,A}_{21:\alpha,j} k^{p/t,A}_{32:\alpha,j} + k^{p/t,A}_{12:\alpha,j} k^{p/t,A}_{23} k^{p/t,A}_{34} + k^{p/t,A}_{14} k^{p/t,A}_{21:\alpha,j} k^{p/t,A}_{34} + k^{p/t,A}_{14} k^{p/t,A}_{23} k^{p/t,A}_{34} \\ & + k^{p/t,A}_{12:\alpha,j} k^{p/t,A}_{23} k^{p/t,A}_{41:\alpha,j} + k^{p/t,A}_{12:\alpha,j} k^{p/t,A}_{23} k^{p/t,A}_{43} + k^{p/t,A}_{14} k^{p/t,A}_{21:\alpha,j} k^{p/t,A}_{43} + k^{p/t,A}_{14} k^{p/t,A}_{23} k^{p/t,A}_{43} \\ & + k^{p/t,A}_{12:\alpha,j} k^{p/t,A}_{32:\alpha,j} k^{p/t,A}_{41:\alpha,j} + k^{p/t,A}_{12:\alpha,j} k^{p/t,A}_{32:\alpha,j} k^{p/t,A}_{43} + k^{p/t,A}_{12:\alpha,j} k^{p/t,A}_{34} k^{p/t,A}_{41:\alpha,j} + k^{p/t,A}_{14} k^{p/t,A}_{32:\alpha,j} k^{p/t,A}_{43} \\ & + k^{p/t,A}_{21:\alpha,j} k^{p/t,A}_{32:\alpha,j} k^{p/t,A}_{41:\alpha,j} + k^{p/t,A}_{21:\alpha,j} k^{p/t,A}_{32:\alpha,j} k^{p/t,A}_{43} + k^{p/t,A}_{21:\alpha,j} k^{p/t,A}_{34} k^{p/t,A}_{41:\alpha,j} + k^{p/t,A}_{23} k^{p/t,A}_{34} k^{p/t,A}_{41:\alpha,j}. \end{aligned} \quad \text{(S30)}$$

N_A is the Avogadro constant (6.022×10^{23} mol^{-1}), $N^{p/t,H}_A$ is the plasma membrane/tonoplast pump density and k's are reaction rates as shown in Figures S1A and S1B. The effects of the apoplastic, cytosolic and vacuolar pH, as well as the trans-plasma membrane and trans-tonoplast potentials, on binding and unbinding are incorporated into the relevant reaction rates,

$$k^{p/t,A}_{41:\alpha,j} = k^{p/t,A,0}_{41} C^{c,H}_{\alpha,j} \quad \text{(S31)}$$

$$k^{p/t,A}_{32:\alpha,j} = k^{p/t,A,0}_{32} C^{a/v,H}_{\alpha,j}, \quad \text{(S32)}$$

$$k^{p/t,A}_{12:\alpha,j} = k^{p/t,A,0}_{12} \exp\left(\frac{F \Delta \psi^{p/t}_{\alpha,j}}{2 R_g T} \right), \quad \text{(S33)}$$

$$k^{p/t,A}_{21:\alpha,j} = k^{p/t,A,0}_{21} \exp\left(\frac{-F \Delta \psi^{p/t}_{\alpha,j}}{2 R_g T} \right). \quad \text{(S34)}$$

$k^{p/t,A,0}_{41}$ ($k^{p/t,A,0}_{32}$) is the value of $k^{p/t,A}_{41}$ ($k^{p/t,A}_{32}$) when $C^{c,H}$ ($C^{a/v,H}$) is 1 mM, and $k^{p/t,A,0}_{12}$ and $k^{p/t,A,0}_{21}$ are the values of the rate constants at zero trans-plasma membrane/trans-tonoplast potential.

S.3.4 Secondary active transmembrane transport via symporters

Transport via the plasma membrane K^+/H^+ symporters and $Cl^-/2H^+$ symporters is also modeled by four state carrier cycles (see Figures S1C and S1D). K^+/Cl^- fluxes through these

symporters are given by,

$$J^{p,K/Cl}_{SK/SCl:\alpha,j} = \frac{N^{p}_{SK/SCl:\alpha,j}A^{i,p}_{\alpha,j}}{N_A D^{p}_{SK/SCl:\alpha,j}} \left(k^{p,SK/SCl}_{12:\alpha,j} k^{p,SK/SCl}_{23} k^{p,SK/SCl}_{34} k^{p,SK/SCl}_{41:\alpha,j} \right.$$
$$\left. - k^{p,SK/SCl}_{14} k^{p,SK/SCl}_{21:\alpha,j} k^{p,SK/SCl}_{32:\alpha,j} k^{p,SK/SCl}_{43:\alpha,j}\right) \tag{S35}$$

where the denominator term is given by,

$$\begin{aligned}
D^{p}_{SK/SCl:\alpha,j} = {} & k^{p,SK/SCl}_{14} k^{p,SK/SCl}_{21:\alpha,j} k^{p,SK/SCl}_{32:\alpha,j} + k^{p,SK/SCl}_{12:\alpha,j} k^{p,SK/SCl}_{23} k^{p,SK/SCl}_{34} \\
& + k^{p,SK/SCl}_{14} k^{p,SK/SCl}_{21:\alpha,j} k^{p,SK/SCl}_{34} + k^{p,SK/SCl}_{14} k^{p,SK/SCl}_{23} k^{p,SK/SCl}_{34} \\
& + k^{p,SK/SCl}_{12:\alpha,j} k^{p,SK/SCl}_{23} k^{p,SK/SCl}_{41:\alpha,j} + k^{p,SK/SCl}_{12:\alpha,j} k^{p,SK/SCl}_{23} k^{p,SK/SCl}_{43:\alpha,j} \\
& + k^{p,SK/SCl}_{14} k^{p,SK/SCl}_{21:\alpha,j} k^{p,SK/SCl}_{43:\alpha,j} + k^{p,SK/SCl}_{14} k^{p,SK/SCl}_{23} k^{p,SK/SCl}_{43:\alpha,j} \\
& + k^{p,SK/SCl}_{12:\alpha,j} k^{p,SK/SCl}_{32:\alpha,j} k^{p,SK/SCl}_{41:\alpha,j} + k^{p,SK/SCl}_{12:\alpha,j} k^{p,SK/SCl}_{32:\alpha,j} k^{p,SK/SCl}_{43:\alpha,j} \\
& + k^{p,SK/SCl}_{12:\alpha,j} k^{p,SK/SCl}_{34} k^{p,SK/SCl}_{41:\alpha,j} + k^{p,SK/SCl}_{14} k^{p,SK/SCl}_{32:\alpha,j} k^{p,SK/SCl}_{43:\alpha,j} \\
& + k^{p,SK/SCl}_{21:\alpha,j} k^{p,SK/SCl}_{32:\alpha,j} k^{p,SK/SCl}_{41:\alpha,j} + k^{p,SK/SCl}_{21:\alpha,j} k^{p,SK/SCl}_{32:\alpha,j} k^{p,SK/SCl}_{43:\alpha,j} \\
& + k^{p,SK/SCl}_{21:\alpha,j} k^{p,SK/SCl}_{34} k^{p,SK/SCl}_{41:\alpha,j} + k^{p,SK/SCl}_{23} k^{p,SK/SCl}_{34} k^{p,SK/SCl}_{41:\alpha,j}.
\end{aligned} \tag{S36}$$

$N^{p}_{SK/SC:\alpha,j}$ is the number of K^+/H^+ ($Cl^-/2H^+$) symporters per unit area of plasma membrane and the rate constants ($k^{p,SK/SCl}$'s) are shown in Figures S1C and S1D. The relevant rate constants include concentration and electric potential dependence,

$$k^{p,SK}_{32:\alpha,j} = k^{p,SK,0}_{32} C^{a,H}_{\alpha,j} \tag{S37}$$

$$k^{p,SK}_{43:\alpha,j} = k^{p,SK,0}_{43} C^{a,K}_{\alpha,j} \tag{S38}$$

$$k^{p,SK}_{41:\alpha,j} = k^{p,SK,0}_{41} C^{c,K}_{\alpha,j} C^{c,H}_{\alpha,j}, \tag{S39}$$

$$k^{p,SK}_{12:\alpha,j} = k^{p,SK,0}_{12} \exp\left(\frac{F\Delta\psi^{p}_{\alpha,j}}{R_g T}\right), \tag{S40}$$

$$k^{p,SK}_{21:\alpha,j} = k^{p,SK,0}_{21} \exp\left(\frac{-F\Delta\psi^{p}_{\alpha,j}}{R_g T}\right), \tag{S41}$$

$$k^{p,SCl}_{32:\alpha,j} = k^{p,SCl,0}_{32} \left(C^{a,H}_{\alpha,j}\right)^2, \tag{S42}$$

$$k^{p,SCl}_{43:\alpha,j} = k^{p,SCl,0}_{43} C^{a,Cl}_{\alpha,j}, \tag{S43}$$

$$k^{p,SCl}_{41:\alpha,j} = k^{p,SCl,0}_{41} C^{c,Cl}_{\alpha,j} \left(C^{c,H}_{\alpha,j}\right)^2, \tag{S44}$$

$$k^{p,SCl}_{12:\alpha,j} = k^{p,SCl,0}_{12} \exp\left(\frac{F\Delta\psi^{p}_{\alpha,j}}{2R_g T}\right), \tag{S45}$$

$$k^{p,SCl}_{21:\alpha,j} = k^{p,SCl,0}_{21} \exp\left(\frac{-F\Delta\psi^p_{\alpha,j}}{2R_gT}\right). \tag{S46}$$

$k^{p,SK/SCl,0}$ is the value of $k^{p,SK/SCl}$ when the relevant ion concentration/s are 1mM, and $k^{p,SK/SCl,0}_{12}$ and $k^{p,SK/SCl,0}_{21}$ are the values of the rate constants at zero plasma membrane electric potential difference ($\Delta\psi^p = 0$).

K^+ fluxes ($J^{p,K}_{SK}$) and H^+ fluxes ($J^{p,H}_{SK}$) through the K^+/H^+ symporter are equal, $J^{p,H}_{SK} = J^{p,K}_{SK} = J^p_{SK}$ (Maathuis et al., 1997); while H^+ fluxes through the Cl^-/2H^+ symport ($J^{p,H}_{SCl}$) are twice the Cl^- fluxes ($J^{p,Cl}_{SCl}$) (Beilby and Walker, 1981; Felle, 1994).

S.3.5 Secondary active transmembrane transport via antiporters

Using the law of mass action and the constraint condition resulting from the electrochemical potential at zero net flux through the antiporter (see Foster and Miklavcic (2015)), Na^+ flux through plasma membrane/tonoplast Na^+/H^+ antiporters is given by,

$$J^{p/t,Na}_{XNa:\alpha,j} = A^{i,p/t}_{\alpha,j} k^{p/t}_{XNa:\alpha,j} \left(C^{c,Na}_{\alpha,j} C^{a/v,H}_{\alpha,j} - C^{a/v,Na}_{\alpha,j} C^{c,H}_{\alpha,j}\right), \tag{S47}$$

and K^+ flux through the tonoplast K^+/H^+ antiporter is given by,

$$J^{t,K}_{XK:\alpha,j} = A^{i,t}_{\alpha,j} k^t_{XK:\alpha,j} \left(C^{c,K}_{\alpha,j} C^{v,H}_{\alpha,j} - C^{v,K}_{\alpha,j} C^{c,H}_{\alpha,j}\right). \tag{S48}$$

$k^{p/t}_{XNa:\alpha,j}$ is a forward reaction rate for transport via plasma membrane/tonoplast Na^+/H^+ antiporters and $k^t_{XK:\alpha,j}$ is a forward reaction rate for transport via tonoplast K^+/H^+ antiporters (Foster and Miklavcic, 2015).

Na^+ and H^+ fluxes through Na^+/H^+ antiporters are equal in magnitude, but opposite in direction $\left(J^{p/t,H}_{XNa:\alpha,j} = -J^{p/t,Na}_{XNa:\alpha,j}\right)$. Similarly, H^+ fluxes through K^+/H^+ antiporters are given by, $J^{t,H}_{XK:\alpha,j} = -J^{t,K}_{XK:\alpha,j}$.

S.3.6 Apoplastic binding of cations to fixed anions

Using the law of mass action, the net rate of cation dissociation from the fixed anions in the apoplast, $R^{bind,n}_{\alpha,j}$, is given by,

$$R^{bind,H}_{\alpha,j} = k^H_{dn} C^{a,HB}_{\alpha,j} - k^H_{an} C^{a,H}_{\alpha,j} C^{a,B}_{\alpha,j}, \tag{S49}$$

$$R^{bind,Na}_{\alpha,j} = k^{Na}_{dn} C^{a,NaB}_{\alpha,j} - k^{Na}_{an} C^{a,Na}_{\alpha,j} C^{a,B}_{\alpha,j}, \tag{S50}$$

$$R^{bind,K}_{\alpha,j} = k^K_{dn} C^{a,KB}_{\alpha,j} - k^K_{an} C^{a,K}_{\alpha,j} C^{a,B}_{\alpha,j}, \tag{S51}$$

$$R^{bind,Cl}_{\alpha,j} = 0, \tag{S52}$$

where $C^{a,B}_{\alpha,j}$ is the concentration of apoplastic anionic charges that are not bound to cations, and $k^n_{an/dn}$ are the binding association/dissociation reaction rates.

S.3.7 Cytosolic and vacuolar H$^+$ buffering

A constant buffer capacity (β) is assumed in cytosols and vacuoles. The change in pH in these compartments is given by (Foster and Miklavcic, 2015, 2017),

$$\frac{dpH_{\alpha,j}^{c/v}}{dt} = -\frac{1}{\beta_{\alpha,j}^{c/v}} \frac{dC_{\alpha,j}^{c/v,H}}{dt}. \tag{S53}$$

$dC_{\alpha,j}^{c/v,H}/dt$ is the unbuffered change in H$^+$ concentration due to transmembrane and symplastic fluxes − found using Eqs. (S11) and (S12) − and pH$_{\alpha,j}^{c/v}$ is the buffered pH which can be used to calculate the concentration of free H$^+$ ($C_{\alpha,j}^{c/v,H_f} = 1000 \times 10^{-pH_{\alpha,j}^{c/v}}$ mM). The concentration of buffering anions in each cytosol/vacuole ($C_{\alpha,j}^{c/v,B}$) is calculated using (Foster and Miklavcic, 2015, 2017),

$$\frac{d}{dt}\left(V_{\alpha,j}^{c/v} C_{\alpha,j}^{c/v,B}\right) = \frac{d}{dt}\left(V_{\alpha,j}^{c/v} C_{\alpha,j}^{c/v,H_f}\right) - \frac{d}{dt}\left(V_{\alpha,j}^{c/v} C_{\alpha,j}^{c/v,H}\right). \tag{S54}$$

S.3.8 Electric potential calculations

The assumption of electroneutrality in each apoplastic, cytosolic and vacuolar compartment leads to a set of zero net current conditions,

$$F\sum_{n=1}^{N} Z^n S_{in,\alpha,j}^{a,rad,n} - F\sum_{n=1}^{N} Z^n S_{out,\alpha,j}^{a,rad,n} + F\sum_{n=1}^{N} Z^n S_{in,\alpha,j}^{a,ax,n} - F\sum_{n=1}^{N} Z^n S_{out,\alpha,j}^{a,ax,n} + N_{\alpha,j}^{cell} F\sum_{n=1}^{N} Z^n S_{in,\alpha,j}^{p,n} = 0, \tag{S55}$$

$$F\sum_{n=1}^{N} Z^n S_{in,\alpha,j}^{s,rad,n} - F\sum_{n=1}^{N} Z^n S_{out,\alpha,j}^{s,rad,n} + F\sum_{n=1}^{N} Z^n S_{in,\alpha,j}^{s,ax,n} - F\sum_{n=1}^{N} Z^n S_{out,\alpha,j}^{s,ax,n} - N_{\alpha,j}^{cells} F\sum_{n=1}^{N} Z^n S_{in,\alpha,j}^{p,n} = 0, \tag{S56}$$

$$F\sum_{n=1}^{N} Z^n S_{in,\alpha,j}^{t,n} = 0. \tag{S57}$$

N represents the total number of charged species under consideration, which varies between compartments. Eqs. (S55) to (S57) represent a system of nonlinear equations, which is solved to find $\psi_{\alpha,j}^{a}$, $\psi_{\alpha,j}^{c}$ and $\psi_{\alpha,j}^{v}$.

S.4. COMPUTATIONAL DETAILS

The nonlinear, coupled, system of differential algebraic equations represented by Eqs. (S3), (S10), (S11), (S12), (S13), (S53), (S54), (S55), (S56) and (S57) was solved numerically in

MATLAB using the ode15s package. The hydraulic pressure was determined by solving the linear system of equations represented by Eqs. (S1) and (S2) at each time step.

A two-stage simulation process was used to represent the typical experimental conditions of a salt free environment (the pre-salt stage) into which NaCl is introduced (the salt stress stage). In the first, pre-salt, stage Na^+ was absent. At the start of the salt stress stage NaCl was introduced into the external medium. The steady-state results of the pre-salt simulation were used as initial conditions for the salt stress stage.

The boundary conditions were (Foster and Miklavcic, 2017):

- a linear hydrostatic pressure gradient in the external medium,
- a constant bulk concentration of ions in the external medium (zero Na^+ for the pre-salt stage and non-zero Na^+ for the salt stress stage),
- zero apoplastic and symplastic flow across the bottom boundary of the root,
- zero symplastic flow across the external medium-epidermis interface,
- zero symplastic flow across the xylem parenchyma-conductive xylem interface,
- zero symplastic flow across the top boundary of the root,
- zero apoplastic flow across the top boundary of the root for all tissue regions except the xylem,
- a constant apoplastic hydraulic pressure and zero apoplastic concentration gradients across the boundary at the top of the xylem.

S.5. ADDITIONAL RESULTS

Table S1: Optimized parameters obtained using the procedure described in Section 2.2 and Figures 3 and 4.

**Parameter**	**Value**	**Units**
**Outer plasma membrane parameters**		
Na^+/H^+ antiporter reaction rate, k^p_{XNa}	2×10^{-7}	$m^4\ mol^{-1}\ s^{-1}$
H^+ pump density, $N^{p,H}_A$	6×10^{15}	m^{-2}
VI NSCC permeability, P^p_{NSCC}	4×10^{-10}	$m\ s^{-1}$
K^+/H^+ symporter density, N^p_{SK}	2×10^{14}	m^{-2}
**Stelar plasma membrane parameters**		
Na^+/H^+ antiporter reaction rate, k^p_{XNa}	3×10^{-7}	$m^4\ mol^{-1}\ s^{-1}$
H^+ pump density, $N^{p,H}_A$	2×10^{15}	m^{-2}
HKT permeability, P_{HKT}	8×10^{-11}	$m\ s^{-1}$
Cl^- channel permeability, $P^{p,Cl}_C$	4×10^{-9}	$m\ s^{-1}$
***sos1* plasma membrane parameters**		
Na^+/H^+ antiporter reaction rate, k^p_{XNa}	4×10^{-8}	$m^4\ mol^{-1}\ s^{-1}$

Table S2: Summary of membrane transport parameters. Refer to Sections S.3.3 and S.3.4, and Figure S1 for detail about H^+ pump and symporter kinetic parameters. Parameters were obtained from the following sources: Foster and Miklavcic (2015)[(a)] Murai-Hatano and Kuwagata (2007)[(b)], Hills et al. (2012)[(c)], Xu et al. (2006)[(d)], Qi and Spalding (2004)[(e)], Reintanz et al. (2002)[(f)], Ivashikina et al. (2001)[(g)], Maathuis and Sanders (1994)[(h)], Diatloff et al. (2004)[(i)], Demidchik and Tester (2002)[(j)], Xue et al. (2011)[(k)], and Zhu and Steudle (1991)[(l)]. Effective diffusion coefficients were calculated using bulk diffusion coefficients from Haynes (2015) and the methods described in Section S.3.1. The symplastic water permeability was estimated using the Hagen-Poiseuille equation. Apoplastic binding parameters were assumed.

Parameter	Value	Units
**Tonoplast parameters**		
Water permeability, L_p^t [(b)]	4×10^{-12}	m s^{-1} Pa^{-1}
Na^+ channel permeability, $P_C^{t,Na}$ [(a)]	2×10^{-9}	m s^{-1}
Cl^- channel permeability, $P_C^{t,Cl}$ [(a)]	1×10^{-9}	m s^{-1}
Na^+/H^+ antiporter reaction rate, k_{XNa}^t [(a)]	6×10^{-8}	m^4 mol^{-1} s^{-1}
K^+/H^+ antiporter reaction rate, k_{XK}^t [(a)]	1×10^{-11}	m^4 mol^{-1} s^{-1}
H^+ pump kinetic parameters[(a),(c)]:		
$N_A^{t,H}$	1.3×10^{16}	m^{-2}
$k_{12}^{t,A,0}$	1×10^3	s^{-1}
$k_{21}^{t,A,0}$	100	s^{-1}
$k_{23}^{t,A}$	1×10^3	s^{-1}
$k_{32}^{t,A,0}$	5×10^6	$m^3 mol^{-1}$ s^{-1}
$k_{34}^{t,A}$	1×10^{11}	s^{-1}
$k_{43}^{t,A}$	1×10^7	s^{-1}
$k_{14}^{t,A}$	1×10^4	s^{-1}
$k_{41}^{t,A,0}$	3×10^6	m^3 mol^{-1} s^{-1}
**Plasma membrane parameters**		
Plasma membrane water permeability, L_p^p [(b)]	2×10^{-13}	m s^{-1} Pa^{-1}
K^+ IRC parameters[(d),(e),(f)]:		
Channel permeability, P_{IRC}	3×10^{-8}	m s^{-1}
Gating charge, Z_g^{IRC}	0.9	
Half activation potential, $\Delta\psi_{50}^{IRC}$	-150	mV
K^+ ORC parameters[(g)]:		
Gating charge, Z_g^{ORC}	1.1	
$K_{ORC}^{50,a}$	13	mM
H^+ pump kinetic parameters[(c)]:		
$k_{12}^{p,A,0}$	2×10^3	s^{-1}
$k_{21}^{p,A,0}$	2×10^2	s^{-1}
$k_{23}^{p,A}$	5×10^4	s^{-1}

Continued on next page

Table S2 – continued from previous page

Parameter	Value	Units
$k_{32}^{p,A,0}$	1×10^5	$m^3\ mol^{-1}\ s^{-1}$
$k_{34}^{p,A}$	500	s^{-1}
$k_{43}^{p,A}$	10	s^{-1}
$k_{14}^{p,A}$	200	s^{-1}
$k_{41}^{p,A,0}$	2×10^6	$m^3\ mol^{-1}\ s^{-1}$
K^+/H^+ symporter parameters**(c),(h)**:		
$k_{12}^{p,SK,0}$	2	s^{-1}
$k_{21}^{p,SK,0}$	0.9	s^{-1}
$k_{23}^{p,SK}$	1×10^4	s^{-1}
$k_{32}^{p,SK}$	1×10^9	$m^3\ mol^{-1}\ s^{-1}$
$k_{34}^{p,SK}$	1×10^5	s^{-1}
$k_{43}^{p,SK}$	4×10^6	$m^3\ mol^{-1}\ s^{-1}$
$k_{14}^{p,SK}$	50	s^{-1}
$k_{41}^{p,SK}$	1×10^8	$m^6\ mol^{-2}\ s^{-1}$
Plasma membrane Cl^-/H^+ symporter parameters**(c)**:		
N_{SCl}^{p}	4×10^{15}	m^{-2}
$k_{12}^{p,SCl,0}$	1×10^3	s^{-1}
$k_{21}^{p,SCl,0}$	50	s^{-1}
$k_{23}^{p,SCl}$	100	s^{-1}
$k_{32}^{p,SCl}$	1×10^{15}	$m^6\ mol^{-2}\ s^{-1}$
$k_{34}^{p,SCl}$	5×10^4	s^{-1}
$k_{43}^{p,SCl}$	100	$m^3\ mol^{-1}\ s^{-1}$
$k_{14}^{p,SCl}$	100	s^{-1}
$k_{41}^{p,SCl}$	1×10^{12}	$m^9\ mol^{-3}\ s^{-1}$
**Outer root plasma membrane parameters**		
Cl^- channel permeability, $P_C^{p,Cl}$**(i),(j)**	9×10^{-10}	$m\ s^{-1}$
K^+ ORC permeability, P_{ORC}**(a)**	8×10^{-9}	$m\ s^{-1}$
VI NSCC parameters**(j)**:		
Binding constant, K_{NSCC}	0.3	mM
Hill coefficient, h	1	
**Stelar plasma membrane parameters**		
K^+/H^+ symporter density, N_{SK}^{p}**(h)**	4×10^{15}	m^{-2}
K^+ ORC permeability, P_{ORC}**(k)**	7×10^{-9}	$m\ s^{-1}$
**Apoplastic parameters**		
Effective apoplastic diffusion coefficients		
$D_e^{a,H}$	9.31×10^{-9}	$m^2\ s^{-1}$

Continued on next page

Table S2 – continued from previous page

Parameter	Value	Units
$D_e^{a,Na}$	1.33×10^{-9}	$m^2\ s^{-1}$
$D_e^{a,K}$	1.96×10^{-9}	$m^2\ s^{-1}$
$D_e^{a,Cl}$	2.03×10^{-9}	$m^2\ s^{-1}$
Axial apoplastic water permeability, $L_{p:j}^{a,ax\mathbf{(l)}}$	$\left(2.5 \times 10^{-10}\right)/l_j^{ax}$	$m\ s^{-1}\ MPa^{-1}$
Radial apoplastic water permeability, $L_{p:\alpha}^{a,rad\mathbf{(l)}}$	$\left(2.5 \times 10^{-10}\right)/d_\alpha$	$m\ s^{-1}\ MPa^{-1}$
Binding association rate, k_{an}	1×10^{-12}	$m^6\ s^{-1}\ mol^{-1}$
Binding dissociation rate, k_{dn}	1×10^{-18}	$m^3\ s^{-1}$
**Symplastic parameters**		
Effective symplastic diffusion coefficients		
$D_e^{s,H}$	8.47×10^{-9}	$m^2\ s^{-1}$
$D_e^{s,Na}$	7.91×10^{-10}	$m^2\ s^{-1}$
$D_e^{s,K}$	1.35×10^{-9}	$m^2\ s^{-1}$
$D_e^{s,Cl}$	1.42×10^{-9}	$m^2\ s^{-1}$
Symplastic water permeability, L_p^s	1.63×10^{-3}	$m\ s^{-1}\ MPa^{-1}$
Symplastic reflection coefficients		
$\sigma^{s,H}$	0.00	
$\sigma^{s,Na}$	0.06	
$\sigma^{s,K}$	0.03	
$\sigma^{s,Cl}$	0.03	
**Buffering parameters**		
Cytoplasmic buffering capacity, $\beta^{c\mathbf{(a)}}$	23	mM/pH unit
Vacuolar buffering capacity, $\beta^{v\mathbf{(a)}}$	30	mM/pH unit

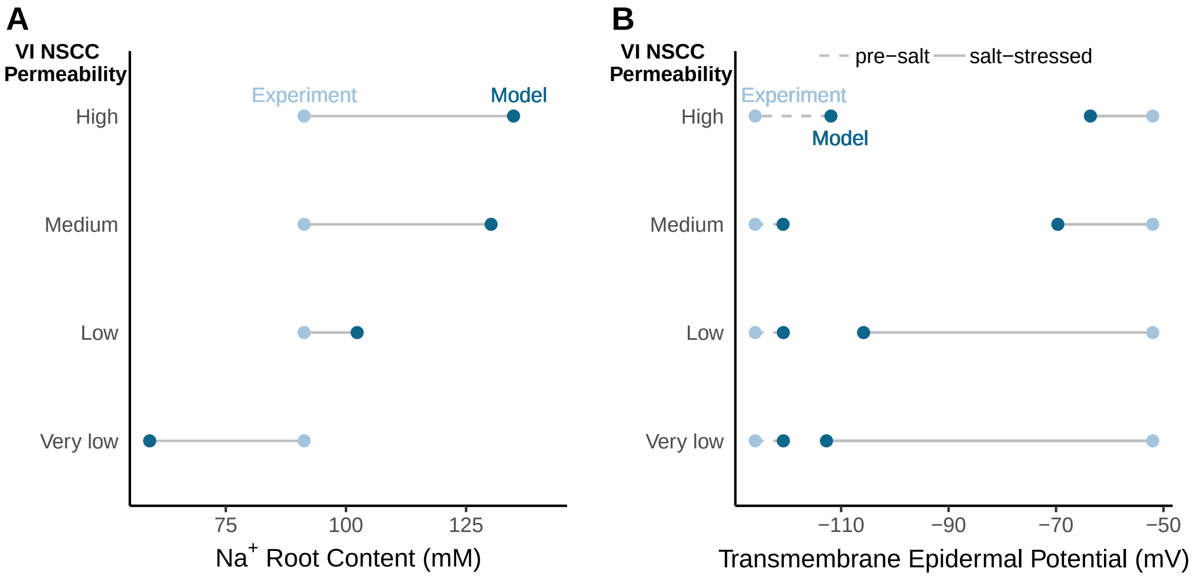

Figure S2. Example of the mismatch between optimized model (dark blue circles) and experimental (light blue circles) **(A)** Na^+ root content, and **(B)** transmembrane epidermal potential for *sos1* scenarios if it is assumed that *sos1* roots have no active plasma membrane Na^+ transporters. Results are shown for plasma membrane voltage insensitive nonselective cation channel (VI NSCC) permeabilities ranging across four orders of magnitude (with each category representing an order of magnitude change in permeability). Experimental data was obtained from Davenport et al. (2007) and Shabala et al. (2005). Comparisons with data from Davenport et al. (2007) were conducted with 50 mM NaCl, 11.9 mM KCl, 0.5 mM Ca^{2+} and a pH of 5.5 in the external medium. Comparisons with data from Shabala et al. (2005) were conducted with 50 mM NaCl, 0.5 mM KCl, 0.1 mM Ca^{2+} and a pH of 5.5 in the external medium.

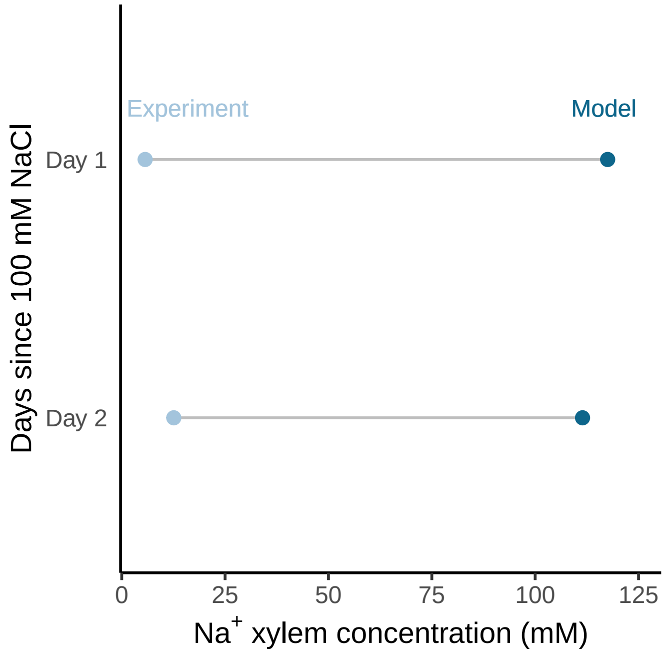

Figure S3. Comparison of experimental (light blue circles) and model (dark blue circles) xylem Na^{+} concentrations in excised roots one and two days after exposure to 100 mM NaCl. Experimental data was obtained from Shi et al. (2002). Simulations were conducted using the parameters optimized using comparisons with intact roots, and with 100 mM NaCl, 1 mM KCl, 0.15 mM Ca^{2+} and a pH of 5.7 in the external medium. Transpiration was switched off in the model to simulate an excised root.

REFERENCES

Beilby, M.J., Walker, N.A., 1981. Chloride transport in *Chara*: I. kinetics and current-voltage curves for a probable proton symport. Journal of Experimental Botany 32, 43–54.

Chowdhury, S., Chanda, B., 2012. Estimating the voltage-dependent free energy change of ion channels using the median voltage for activation. The Journal of General Physiology 139, 3–17.

Davenport, R.J., Muñoz Mayor, A., Jha, D., Essah, P.A., Rus, A.N.A., Tester, M., 2007. The Na^+ transporter AtHKT1;1 controls retrieval of Na^+ from the xylem in *Arabidopsis*. Plant, Cell & Environment 30, 497–507. doi:10.1111/j.1365-3040.2007.01637.x.

Dechadilok, P., Deen, W.M., 2006. Hindrance factors for diffusion and convection in pores. Industrial & Engineering Chemistry Research 45, 6953–6959.

Demidchik, V., Tester, M., 2002. Sodium fluxes through nonselective cation channels in the plasma membrane of protoplasts from *Arabidopsis* roots. Plant Physiology 128, 379–387.

Diatloff, E., Roberts, M., Sanders, D., Roberts, S.K., 2004. Characterization of anion channels in the plasma membrane of *Arabidopsis* epidermal root cells and the identification of a citrate-permeable channel induced by phosphate starvation. Plant Physiology 136, 4136–4149.

Dolan, L., Janmaat, K., Willemsen, V., Linstead, P., Poethig, S., Roberts, K., Scheres, B., 1993. Cellular organisation of the *Arabidopsis thaliana* root. Development 119, 71–84.

Felle, H.H., 1994. The H^+/Cl^- symporter in root-hair cells of *Sinapis alba* (an electrophysiological study using ion-selective microelectrodes). Plant Physiology 106, 1131–1136.

Foster, K.J., Miklavcic, S.J., 2015. Toward a biophysical understanding of the salt stress response of individual plant cells. Journal of Theoretical Biology 385, 130–142.

Foster, K.J., Miklavcic, S.J., 2017. A comprehensive biophysical model of ion and water transport in plant roots. I. Clarifying the roles of endodermal barriers in the salt stress response. Frontiers in Plant Science 8. doi:10.3389/fpls.2017.01326.

Haynes, W.M., 2015. CRC Handbook of Chemistry and Physics. 96 ed., CRC press, Boca Raton.

Hills, A., Chen, Z.H., Amtmann, A., Blatt, M.R., Lew, V.L., 2012. OnGuard, a computational platform for quantitative kinetic modeling of guard cell physiology. Plant Physiology 159, 1026–1042.

van der Horst, H.C., Timmer, J.M.K., Robbertsen, T., Leenders, J., 1995. Use of nanofiltration for concentration and demineralization in the dairy industry: Model for mass transport. J. Membrane Sci. 104, 205–218.

Ivashikina, N., Becker, D., Ache, P., Meyerhoff, O., Felle, H.H., Hedrich, R., 2001. K^+ channel profile and electrical properties of *Arabidopsis* root hairs. FEBS Letters 508, 463–469.

Johansson, I., Wulfetange, K., Porée, F., Michard, E., Gajdanowicz, P., Lacombe, B., Sentenac, H., Thibaud, J.B., Mueller-Roeber, B., Blatt, M.R., Dreyer, I., 2006. External K^+ modulates the activity of the *Arabidopsis* potassium channel SKOR via an unusual mechanism. The Plant Journal 46, 269–281. doi:doi:10.1111/j.1365-313X.2006.02690.x.

Keener, J., Sneyd, J., 2009. Mathematical Physiology I: Cellular Physiology. 2 ed., Springer, New York.

Liesche, J., Schulz, A., 2013. Modeling the parameters for plasmodesmal sugar filtering in active symplasmic phloem loaders. Frontiers in Plant Science 4, 1–11.

Maathuis, F.J., Sanders, D., 1994. Mechanism of high-affinity potassium uptake in roots of *Arabidopsis thaliana*. Proceedings of the National Academy of Sciences of the United States of America 91, 9272–9276.

Maathuis, F.J.M., Sanders, D., Gradmann, D., 1997. Kinetics of high-affinity K^+ uptake in plants, derived from K^+-induced changes in current-voltage relationships. Planta 203, 229–236.

Murai-Hatano, M., Kuwagata, T., 2007. Osmotic water permeability of plasma and vacuolar membranes in protoplasts I. High osmotic water permeability in radish (*Raphanus sativus*) root cells as measured by a new method. Journal of Plant Research 120, 175–189. doi:10.1007/s10265-007-0072-5.

Qi, Z., Spalding, E.P., 2004. Protection of plasma membrane K^+ transport by the salt overly sensitive1 Na^+-H^+ antiporter during salinity stress. Plant Physiology 136, 2548–2555. doi:10.1104/pp.104.049213.

Reintanz, B., Szyroki, A., Ivashikina, N., Ache, P., Godde, M., Becker, D., Palme, K., Hedrich, R., 2002. AtKC1, a silent *Arabidopsis* potassium channel α-subunit modulates root hair K^+ influx. Proceedings of the National Academy of Sciences 99, 4079–4084. doi:10.1073/pnas.052677799.

Shabala, L., Cuin, T.A., Newman, I.A., Shabala, S., 2005. Salinity-induced ion flux patterns from the excised roots of *Arabidopsis sos* mutants. Planta 222, 1041–1050.

Shi, H., Quintero, F.J., Pardo, J.M., Zhu, J.K., 2002. The putative plasma membrane Na^+/H^+ antiporter SOS1 controls long-distance Na^+ transport in plants. The Plant Cell Online 14, 465–477.

Xu, J., Li, H.D., Chen, L.Q., Wang, Y., Liu, L.L., He, L., Wu, W.H., 2006. A protein kinase, interacting with two calcineurin B-like proteins, regulates K^+ transporter AKT1 in *Arabidopsis*. Cell 125, 1347–1360. doi:https://doi.org/10.1016/j.cell.2006.06.011.

Xue, S., Yao, X., Luo, W., Jha, D., Tester, M., Horie, T., Schroeder, J.I., 2011. AtHKT1;1 mediates Nernstian sodium channel transport properties in *Arabidopsis* root stelar cells. PLoS ONE 6, e24725. doi:10.1371/journal.pone.0024725.

Zhu, G.L., Steudle, E., 1991. Water transport across maize roots: Simultaneous measurement of flows at the cell and root level by double pressure probe technique. Plant Physiology 95, 305–315.
